# Phosphonodithioester–Amine Coupling as a Key Reaction Step for the Design of Cationic Amphiphiles Used for Gene Delivery

**DOI:** 10.3390/molecules26247507

**Published:** 2021-12-11

**Authors:** Montassar Khalil, Alexis Hocquigny, Mathieu Berchel, Tristan Montier, Paul-Alain Jaffrès

**Affiliations:** 1 Univ Brest, CNRS, CEMCA UMR 6521, 6 Avenue Victor Le Gorgeu, 29238 Brest, France; Montassar.Khalil@univ-brest.fr (M.K.); mathieu.berchel@univ-brest.fr (M.B.); 2 Univ Brest, INSERM, EFS, UMR 1078, GGB-GTCA, 29200 Brest, France; Alexis.Hocquigny@univ-brest.fr (A.H.); montier@univ-brest.fr (T.M.); 3 CHRU de Brest, Service de Génétique Médicale et Biologie de la Reproduction, Centre de Référence des Maladies Rares “Maladies Neuromusculaires”, 29200 Brest, France

**Keywords:** cationic lipids, phospholipids, click reaction, liposomes, nucleic acids delivery

## Abstract

A convergent synthesis of cationic amphiphilic compounds is reported here with the use of the phosphonodithioester–amine coupling (PAC) reaction. This versatile reaction occurs at room temperature without any catalyst, allowing binding of the lipid moiety to a polar head group. This strategy is illustrated with the use of two lipid units featuring either two oleyl chains or two-branched saturated lipid chains. The final cationic amphiphiles were evaluated as carriers for plasmid DNA delivery in four cell lines (A549, Calu3, CFBE and 16HBE) and were compared to standards (BSV36 and KLN47). These new amphiphilic derivatives, which were formulated with DOPE or DOPE-cholesterol as helper lipids, feature high transfection efficacies when associated with DOPE. The highest transfection efficacies were observed in the four cell lines at low charge ratios (CR = 0.7, 1 or 2). At these CRs, no toxic effects were detected. Altogether, this new synthesis scheme using the PAC reaction opens up new possibilities for investigating the effects of lipid or polar head groups on transfection efficacies.

## 1. Introduction

**Figure 1:**
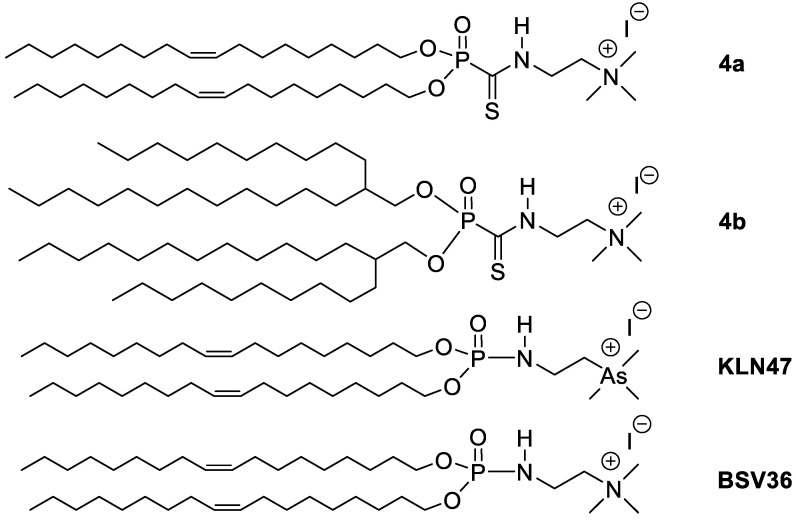
Chemical structure of the two new cationic amphiphiles **4a–b** reported in this work and the structure of **KLN47** and **BSV36,** which were used as references for the transfection experiments.

Cationic amphiphilic compounds form liposomes in water and can interact with nucleic acids (e.g., pDNA, siRNA, mRNA) to produce lipoplexes after full reorganization of the liposomal supramolecular assemblies [1]. This self-reorganization occurs thanks to both ionic interactions and the self-assembly of their lipid chains, limiting their interactions with water [2]. Since the pioneering works of Felgner [3], many cationic amphiphilic structures have been proposed [4], with variations in the structure of the linker [5], the lipid chains [6] or the polar head group [7]. The final goal of all these cationic amphiphiles consists of producing supramolecular assemblies featuring tunable stability in order to be sufficiently stable during the carrying process, while also permitting cargo release in the cytosol after cell internalization, which usually occurs via an endocytosis pathway [8]. Accordingly, different types of liposomes responding to variations in pH [9,10], to reducing agents [11,12] or to enzymatic reactions [13] have been designed. These amphiphilic compounds have been used for in vitro or in vivo nucleic acid delivery assays (pDNA [14], mRNA [15]) aiming to address a broad panel of applications including vaccination [16], tendon healing [17], lung transfection [18,19] or cancer therapy [20,21]. Other type of cationic amphiphiles have been designed with the aim of controlling their molecular shape, which is, according to the Israelachvili theory [22], a key parameter for controlling the type of supramolecular self-assembly. Following this line, we have reported the synthesis of phospholipids using commercially available branched lipid chains [23] or including branched lipid chains prepared via the photo-thiol-ene reaction [24]. These cationic amphiphiles form inverted hexagonal aggregates that promote lipid fusion and favor the transfection process. With the aim of tuning the stability of the supramolecular assemblies, we have also incorporated a thiophosphoramide moiety in order to adjust the robustness of the hydrogen bonds networks present in liposomes and lipoplexes [25]. One important feature for the design of amphiphilic compounds is the versatility of their synthesis in order to explore new structures and to evaluate their transfection capacity. Moreover, the simplicity of the synthesis (e.g., limiting the number of steps) is a key parameter for facilitating the translation of in vitro to in vivo studies via the production of amphiphilic compounds at a multi-gram scale [26]. Accordingly, a convergent synthesis that allows for a better synthesis efficacy is highly desired. With regard to this, the coupling of the lipid moieties to the polar head group in the final step or close to the final step is an attractive strategy. Different click reactions including thiol-ene [27], thiol-yne [28] or copper alkyne-azide coupling—CuAAC [29] have been previously reported for construction of amphiphilic compounds used to prepare vesicles, including some of that were used for transfection. We report here a new convergent synthesis scheme for the preparation of cationic amphiphiles, in which the key step is a phosphonodithioester–amine coupling reaction (PAC reaction). The PAC reaction was initially reported by Masson et al., 1994 [30] as a new method for the preparation of phosphonothioamide. Following this, two studies reported its application for the functionalization of chitosan [31] and for the functionalization of the surface of liposomes [32]. This reaction was also extended to fluorinated phosphodithioacetate [33]. The interest of this reaction comes from its achievement under mild conditions and without any catalyst. We report here for the first time the use of the PAC reaction for the preparation of two cationic amphiphilic compounds **4a–b** (Figure 1) that were used for in vitro transfection assays in four cell lines (A549, Calu3, CFBE, 16HBE). The transfection efficacies were compared to two reference compounds **KLN47** and **BSV36** (Figure 1), permitting us to conclude both the influence of the lipid chain and the effect of the phosphono-thioamide moiety on the transfection efficacy.

## 2. Materials and Methods

### 2.1. Materials

All reagents, including oleyl alcohol (technical grade, 85%) and 2-decyl-tetradecan-1-ol (97%), were purchased from commercial sources and used as received (Aldrich, TCI). Solvents (THF, CH_2_Cl_2_) were dried with an MBRAUN solvent purification system and kept under a molecular sieve. Milli-Q water was used as solvent for the liposomal formulations. NMR spectroscopy data were recorded with a Bruker Avance 400 (^1^H: 400.0 MHz, ^31^P: 161.9 MHz, T = 293 K) or a Bruker Avance 500 (^13^C: 125.7 MHz, T = 293 K). The ^13^C NMR spectra were recorded with a J-MOD sequence (J-modulated spin-echo). The chemical shifts are reported in δ[ppm] (multiplicity, coupling constant J, number of protons) relative to the solvent residual signal as the internal standard. For ^31^P NMR, an external reference (H_3_PO_4_; δ = 0 ppm) was used. The coupling constants are given in Hertz [Hz]. Mass spectra were recorded with the autoflex III Brucker MALDI-TOF mass spectrometer (α-Cyano-4-hydroxycinnamic acid (HCCA) or a SYNAPT XS High Resolution Mass Spectrometer (ESI-qTOF). IR were recorded using attenuated total reflection mode (ATR) on a Bruker platinum ATR VERTEX 70 IR spectrometer. The mean particle diameter and zeta potential (ξ) were measured using a Malvern Zetasizer Nano ZS at 25 °C using 1 cm disposable polystyrene cuvettes. **KLN47** [34] and **BSV36 [35]** were synthesized following reported procedures.

### 2.2. Synthesis and Characterization

**Figure 2:**
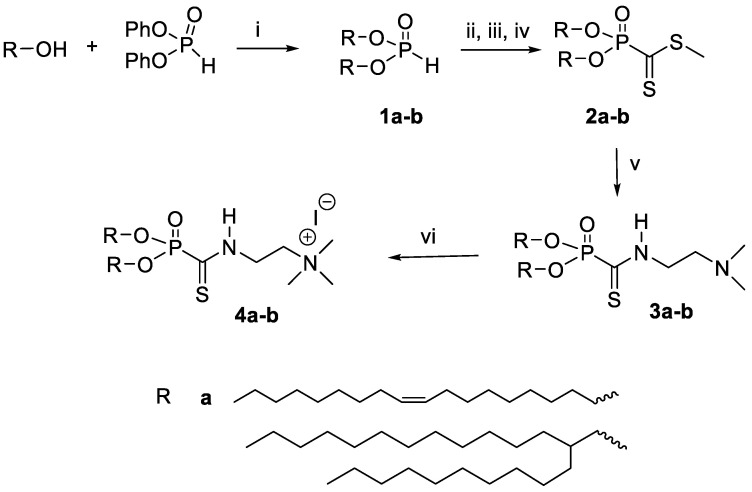
Synthesis of cationic amphiphiles. (**i**) Heating at 140 °C under vacuum in a Kugelrohr distillation apparatus for 4 h; (**ii**) NaH, anhydrous THF, reflux, 4 h; (**iii**) addition of CS_2_ at −78 °C, then warmed up and stirred at RT for 2 h; (**iv**) CH_3_I; (**v**) *N*,*N*-dimethylethylenediamine; (**vi**) CH_3_I, RT, 2 h.

The synthesis scheme is reported in Figure 2. This sequence starts with the synthesis of lipid-phosphonodithioformiates **2a–b** in two steps involved in the PAC reaction to yield **3a–b**. Then, in the final step, the methylation of the tertiary amine produced the cationic amphiphiles **4a–b**. The NMR spectra are accessible in the supporting information (SI).

#### 2.2.1. Dioleylphosphite **1a**

A mixture of oleyl alcohol (3 g, 11.2 mmol) and diphenylphosphite (1.25 g, 5.3 mmol) was placed in a Kugelrohr distillation apparatus. The mixture was heated at 140 °C under reduced pressure (4.10^−2^ mbar) for 4 h. Phenol, which was sublimed during this heating period, was discarded. Then, the temperature was increased to 180 °C (4.10^−2^ mbar) for 1 h to remove the excess oleyl alcohol. The undistilled compound **1a** was isolated as a pale-yellow oil (3.0 g; 97% yield). ^1^H NMR (400.0 MHz, CDCl_3_): δ = 6.79 (d, ^1^*J_HP_* = 692, 1H), 5.36–5.33 (m, 4H), 4.09–4.03 (m, 4H), 2.03–1.98 (m, 8H), 1.70–1.66 (m, 4H), 1.30–1.25 (m, 49H), 0.88 (t, ^3^*J_HH_* = 6.9, 6H); ^31^P{^1^H} NMR (161.9 MHz, CDCl_3_): δ = 9.12; ^13^C{^1^H} J-MOD NMR (125.7 MHz, CDCl_3_): δ = 132.70 and 132.45 (2C, H**C**=**C**H), 68.51 (d, ^2^*J_CP_* = 5.7, **C**H_2_-O), 35.32 (CH_2_), 34.62 (CH_2_), 33.13 (d, ^3^*J_CP_* = 6.0, **C**H_2_-CH_2_-O), 32.48–31.81 (CH_2_ fatty chains), 29.93 (CH_2_), 29.89 (CH_2_), 28.21 (CH_2_), 25.39 (CH_2_), 16.82 (CH_3_).

#### 2.2.2. Bis(2-decyltetradecyl)phosphite **1b**

The same protocol used for **1a** was applied. Diphenylphosphite (0.99 g, 4.2 mmol), 2-decyltetradecan-1-ol (3.00 g, 8.45 mmol). Compound **1b** was isolated as a colorless oil (3.0 g; 94% yield). ^1^H NMR (400.0 MHz, CDCl_3_): δ = 6.81 (d, ^1^*J_HP_* = 691, 1H), 4.00–3.97 (m, 4H), 1.65–1.64 (m, 4H), 1.34–1.24 (m, 83H), 0.91 (t, *J* = 6.8, 12H); ^31^P{^1^H} NMR (161.9 MHz, CDCl_3_): δ = 9.61; ^13^C{^1^H} J-MOD NMR (125.7 MHz, CDCl_3_): δ = 70.80 (d, ^2^*J_CP_* = 6.1, **C**H_2_-O), 41.41 (d, ^3^*J_CP_* = 6.5, **C**H-CH_2_-O), 34.64 (CH_2_), 33.43 (CH_2_), 32.66 (CH_2_), 32.38–32.33 (**C**H_2_ fatty chains), 32.07 (CH_2_), 29.36 (CH_2_), 25.40 (CH_2_), 16.80 (CH_3_); Maldi TOF (matrix: HCCA): [M+Na]^+^ calculated for [C_48_H_99_O_3_P+Na]^+^ = 777.72; observed [M+Na]^+^ = 777.82.

#### 2.2.3. Methyl Dioleylphosphonodithioformate **2a**

To a mixture of NaH (107 mg, 4.46 mmol) in 15 mL of anhydrous tetrahydrofuran placed in a Schlenk flask under nitrogen, a solution of phosphite **1a** (2 g, 3.43 mmol) solubilized in 15 mL of anhydrous THF was added under stirring at room temperature. At the end of the addition, the mixture was heated to 54 °C for 4 h (a limpid solution was formed). The ^31^P NMR monitoring (D_2_O in capillary used as an internal standard) revealed a peak at 152 ppm for the phosphite sodium salt. Then, the solution was cooled down to −78 °C and treated with anhydrous carbon disulfide (1.56 g, 1.25 mL, 20.6 mmol). The mixture was stirred for 2 h at room temperature and methyl iodide (536 mg, 3.77 mmol) was subsequently added. After stirring for an additional 2 h, the mixture was washed with a saturated aqueous solution of ammonium chloride and was extracted twice with 40 mL of ethyl acetate. The organic phase was washed with brine, dried over anhydrous sodium sulfate, filtrated and concentrated. A purification by chromatography on silica gel (eluent of *n*-hexane/ethyl acetate from 100:0 to 70:30 in volume) gave **2a** as a purple oil, obtained with a 20% yield (450 mg). ^1^H NMR (400.0 MHz, CDCl_3_): δ = 5.38–5.35 (m, 4H), 4.24–4.16 (m, 4H), 2.72 (s, 3H), 2.04–2.01 (m, 8H), 1.75–1.69 (m, 4H), 1.29–1.28 (m, 56H), 0.90 (t, ^3^*J_HH_* = 6.8, 6H); ^31^P{^1^H} NMR (161.9 MHz, CDCl_3_): δ = −1.69; ^13^C{^1^H} J-MOD NMR (125.7 MHz, CDCl_3_): δ = 229.58 (d, ^1^*J_CP_* = 176.6, **C**=S), 129.96 + 129.74 (CH_2_-**C**H=**C**H-CH_2_), 68.57 (d, ^2^*J_CP_* = 6.8, **C**H_2_-O-P), 31.91 to 22.68 (**C**H_2_ fatty chains), 19.20 (S-**C**H_3_), 14.11 (**C**H_3_-CH_2_); Maldi TOF (matrix: HCCA): [M+Na]^+^ calculated for [C_38_H_73_O_3_PS_2_Na]^+^ = 695.46; observed [M+Na]^+^ = 695.72.

#### 2.2.4. Methyl Bis-(2-decanyltetradecyl)phosphonodithioformate **2b**

Same protocol as for **2a**. NaH (83 mg, 3.44 mmol), phosphite **1b** (2 g, 2.65 mmol). Intermediate ^31^P NMR (D_2_O in a capillary used as an internal standard): 152 ppm; carbon disulfide (1.21 g, 960 µL, 15.9 mmol), methyl iodide (414 mg, 180 µL, 2.92 mmol). Purification by chromatography on silica gel (eluent *n*-hexane/ethyl acetate: 100:0 to 70:30). Compound **2b** was isolated in 20% yield (400 mg). ^1^H NMR (400.0 MHz, CDCl_3_): δ = 4.12–4.09 (m, 4H), 2.72 (s, 3H), 1.67 (m, 2H), 1.33–1.28 (m, 93H), 0.91 (t, ^3^*J_HH_* = 6.8, 13H); ^31^P{^1^H} NMR (161.9 MHz, CDCl_3_): δ = −1.85; ^13^C{^1^H} J-MOD NMR (125.7 MHz, CDCl_3_): δ = 229.64 (d, ^1^*J_CP_* = 177.0, **C**=S), 70.91 (d, ^2^*J_CP_* = 7.4, **C**H_2_-O-P), 38.74 (d, ^3^*J_CP_* = 6.4, **C**H-CH_2_-O-P), 31.95 to 22.71 (**C**H_2_, fatty chains), 19.11 (S-**C**H_3_), 14.12 (**C**H_3_-CH_2_); Maldi TOF (matrix: HCCA): [M+Na]^+^ calculated for [C_50_H_101_O_3_PS_2_Na]^+^ = 867.68; observed [M+Na]^+^ =867.87.

#### 2.2.5. Dioleyl ((2-(dimethylamino)ethyl)carbamothioyl)phosphonate **3a**

To a solution of **2a** (200 mg, 0.297 mmol, 1 eq.) in 10 mL of CHCl_3_, *N*,*N*-dimethylethylenediamine (28.81 mg, 35 µL, 0.326 mmol, 1.1 eq.) was slowly added. The reaction was stirred at room temperature for 2 h. The excess amine was removed under vacuum. The compound was purified by chromatography on silica gel (CH_2_Cl_2_/MeOH: 90/10 (*v*/*v*)) to produce **3a** as a yellow wax (121 mg; 57% yield). ^1^H NMR (400.0 MHz, CDCl_3_): δ = 5.37–5.32 (m, 4H), 4.21–4.13 (m, 4H), 3.69 (t, ^3^*J_HH_* = 4.6, NH-CH_2_-, 2H), 2.60 (t, ^3^*J_HH_* = 5.9, N(CH_3_)_2_-CH_2_-, 2H), 2.27 (s, 6H), 2.01–1.98 (m, 8H), 1.71–1.68 (m, 4H), 1.36–1.25 (m, 52H), 0.87 (t, ^3^*J_HH_* = 6.8, 6H); ^31^P{^1^H} NMR (161.9 MHz, CDCl_3_): δ = −1.20, ^13^C{^1^H} J-MOD NMR (125.7 MHz, CDCl_3_): δ = 193.07 (d, ^1^*J_CP_* = 182.4, **C**=S), 129.98 + 129.77 (CH_2_-**C**H=**C**H-CH_2_), 68.87 (d, ^2^*J_CP_* = 7.0, **C**H_2_-O-P), 55.67 (**C**H_2_-N(CH_3_)_2_), 44.95 (N(**C**H_3_)_2_-CH_2_-), 42.39 (d, ^3^*J_CP_* = 7.85, P-CS-NH-**C**H_2_-), 31.91 to 22.69 (m, **C**H_2_ fatty chains), 14.12 (**C**H_3_-CH_2_); Maldi TOF (matrix: HCCA): [M+H]^+^ calculated for [C_41_H_82_N_2_O_3_PS]^+^ = 713.58; observed [M+H]^+^ =713.74.

#### 2.2.6. Bis(2-decanyltetradecyl) ((2-(dimethylamino)ethyl)carbamothioyl)phosphonate **3b**

Same protocol as for **3a**. **2b** (100 mg, 0.118 mmol, 1 eq.), *N*,*N*-dimethylethylenediamine (11.47 mg, 14 µL, 0.13 mmol, 1.1 eq.). After purification by chromatography on silica gel (CH_2_Cl_2_/MeOH: 90/10 (*v*/*v*)), **3b** was isolated as a yellow wax (51 mg, 49% yield). ^1^H NMR (400.0 MHz, CDCl_3_): δ = 4.10–4.04 (m, 4H), 3.67 (t, ^3^*J_HH_* = 5.9, NH-CH_2_-, 2H), 2.57 (t, ^3^*J_HH_* = 5.62, N(CH_3_)_2_-CH_2_-, 2H), 2.25 (s, 6H), 1.62 (m, 2H), 1.29–1.24 (m, CH_2_ fatty chains), 0.87 (t, ^3^*J_HH_* = 6.8, 12H); ^31^P{^1^H} NMR (161.9 MHz, CDCl_3_): δ = −1.66; **^1^**^3^C{^1^H} J-MOD NMR (125.7 MHz, CDCl_3_): δ = 192.95 (d, ^1^*J_CP_* = 182.9, **C**=S), 71.23 (d, ^2^*J_CP_* = 7.5, **C**H_2_-O-P), 65.70 (-**C**H_2_-N(CH_3_)_3_-), 55.67 (**C**H_2_-NH), 44.98 (N(**C**H_3_)_3_-), 38.71 (d, *^3^J_CP_* = 6.3, **C**H-CH_2_-O-P), 31.93 to 29.37 (m, **C**H_2_ fatty chains), 22.69 (-**C**H_2_-CH_3_) 14.11 (CH_2_-**C**H_3_); Maldi TOF (matrix: HCCA): [M+H]^+^ calculated for [C_53_H_110_N_2_O_3_PS]^+^ = 885.80; observed [M+H]^+^ = 885.93.

#### 2.2.7. 2-((Dioleyloxyphosphoryl)methanethioamido)-N,N,N-trimethylethan-1-aminium iodide **4a**

Methyl iodide (200 mg, 1.4 mmol, 86 µL, 10 eq.) was added to a solution of **3a** (100 mg, 0.14 mmol, 1 eq.) in CHCl_3_ (5 mL). The solution was stirred overnight at 20 °C. The solvent and the excess of methyl iodide were evaporated under vacuum. After purification by chromatography on silica gel (CH_2_Cl_2_/MeOH: 100/0 to 90/10 (*v*/*v*)), **4a** was isolated as a yellow wax (76 mg; 75% yield).^1^H NMR (400.0 MHz, CDCl_3_): δ = 5.32–5.29 (m, 4H), 4.38 (C**H_2_**-N(CH_3_)_3_, 2H), 4.18–4.15 (m, CH_2_-O-P, 4H), 4.00 (t, ^3^*J_HH_* = 6.3, NH-C**H_2_**-, 2H), 3.45 (s, N(CH_3_)_3_, 9H), 1.98–1.95 (m, 4H), 1.70–1.67 (m, 4H), 1.25–1.22 (m, fatty chains), 0.84 (t, ^3^*J_HH_* = 6.8, 6H); ^31^P{^1^H} NMR (161.9 MHz, CDCl_3_): δ = −2.34; ^13^C{^1^H} J-MOD NMR (125.7 MHz, CDCl_3_): δ = 196.18 (d, ^1^*J_CP_* = 186.0, **C**=S), 130.01 + 129.77 (CH_2_-**C**H=**C**H-CH_2_), 69.42 (d, ^2^*J_CP_* = 7.0, **C**H_2_-O-P), 62.83 (**C**H_2_-N(CH_3_)_3_), 54.63 (N(**C**H_3_)_3_-), 38.82 (d, ^3^*J_CP_* = 8.6, P-CS-NH-**C**H_2_-), 29.80 to 27.25 (m, **C**H_2_ fatty chains), 14.15 (**C**H_3_-CH_2_).

#### 2.2.8. 2-((Bis(2-decanyltetradecyloxy)phosphoryl)methanethioamido)-N,N,N-trimethylethan-1-ammonium iodide **4b**

Same protocol as for **4a**. **3b** (51 mg, 0.058 mmol, 1 eq.), methyl iodide (82 mg, 0.576 mmol, 35 µL, 10 eq). After purification by chromatography on silica gel (CH_2_Cl_2_/MeOH: 90/0 to 10 (*v*/*v*)), **4a** was isolated as a wax (44 mg; 85% yield). ^1^H NMR (400.0 MHz, CDCl_3_): δ = 4.42 (t, *^3^J_HH_* = 5.9, NH-CH_2_-, 2H), 4.12–4.05 (m, 4H), 4.03 (t, ^3^*J_HH_* = 6.4, N(CH_3_)_2_-CH_2_-, 2H), 3.48 (s, 9H), 1.81 (m, 2H), 1.68 (m, 2H), 1.30–1.25 (m, CH_2_ fatty chains), 0.87 (t, ^3^*J_HH_* = 6.9, 12H); ^31^P{^1^H} NMR (161.9 MHz, CDCl_3_): δ = −2.24; ^13^C{^1^H} J-MOD NMR (125.7 MHz, CDCl_3_): δ = 196.24 (d, ^1^*J_CP_* = 184.5, **C**=S), 72.03 (d, ^2^*J_CP_* = 7.5, **C**H_2_-O-P), 62.68 (-**C**H_2_-N(CH_3_)_3_-), 54.62 (N(**C**H_3_)_3_-), 38.73 (d, ^3^*J_CP_* = 6.1, **C**H-CH_2_-O-P), 31.95 (NH-**C**H_2_-), 30.55 to 29.41 (m, **C**H_2_ fatty chains), 14.11 (CH_2_-**C**H_3_); HRMS (ESI-qTOF): *m/z* calculated for [C_54_H_112_N_2_O_3_PS]^+^ [M]^+^ = 899.8131; observed [M]^+^ = 899.8121.

### 2.3. Preparation of Liposomes

The cationic lipids **BSV36** and **KLN47** (used as references) were formulated as previously reported [35]. The new compounds **4a** and **4b** were formulated as liposomal solutions with a helper lipid—1,2-Dioleoyl-*sn*-glycero-3-phosphoethanolamine (DOPE) and cholesterol (Chol)—by hydration of a lipid film or by ethanolic injection, as summarized in Table 1. Briefly, for LF1–2 formulations (1.5 µmol of **4a** or **4b**), **4a** was taken from a stock solution in chloroform and a colipid (1.5 µmol of DOPE or/and cholesterol taken from a stock solution in chloroform) were placed in a 10 mL round-bottom flask. The chloroform was evaporated under vacuum with a rotary evaporator. The resulting lipid film was hydrated with 1 mL of water (MilliQ water) and left at 4 °C for one night. Then, the solution was vortexed and sonicated (30 min at 45 °C.) to produce a transparent solution.

For LF3 formulation, the ethanolic injection method was applied because the method using the hydration of a lipid film was unsuccessful. Briefly, a mixture of **4b** (1.5 µmol) and DOPE (1.5 µmol) in a small volume of ethanol (1% of the final volume of the liposomal solution) was slowly added to an aqueous solution (1 mL) under stirring. In the final step, ethanol was removed by evaporation at 45 °C under vacuum.

### 2.4. Size and Zeta Measurements

An aliquot of 50 µL of the liposomal solutions was diluted in 950 µL of Milli-Q water. This solution was transferred into a MALVERN DTS1060 cuvette for measurements.

The same procedure was applied to record the size and zeta potential of the lipoplexes at different charge ratios (CRs). A total of 0.625 μg of plasmid pGM144 was used to prepare 50 μL of complex solutions.

### 2.5. DNA Complexation

This procedure was similar to a previously reported protocol [36]. Liposomes were sonicated for 10 min at 38 °C before use, while the concentration of the pDNA solution was quantified with the NanoDrop ND-1000. Briefly, lipoplexes were prepared by mixing pDNA (0.25 µg pGM144 for 20 µL of complexes solutions; pGM144 is a plasmid coding for luciferase) with each liposomal solution in water at different charge ratios (CRs) from 0.7 to 4 (CRs are defined by the ratio of the number of cationic charges coming from the cationic amphiphiles divided by the number of negative charges coming from the phosphate moieties of the plasmid). All mixtures were incubated at room temperature for 30 min. The complexes were subjected to electrophoresis in 0.8% agarose gel at 100 V, 90 mA for 20 min. The gel was stained with BET (ethidium bromide) and then visualized using a UV transilluminator.

**Figure 3:**
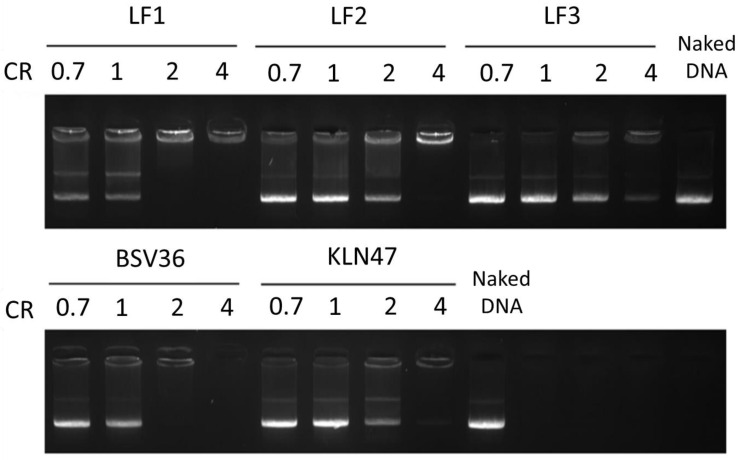
Gel electrophoresis of complexes prepared by mixing LF1-3, BSV36, KLN47 and pGM144 at CRs ranging from 0.7 to 4 in water.

As shown in Figure 3, the DNA complexation of LF1–LF3 requires a charge ratio of 2 or 4 to obtain a full complexation of pDNA. When partial complexation is observed (CR = 0.7, 1 and 2), the intensity of the free DNA appears to correlate with the CR, with progressive complexation observed with increases in the CR.

### 2.6. In Vitro Transfection Efficacies

**Figure 4:**
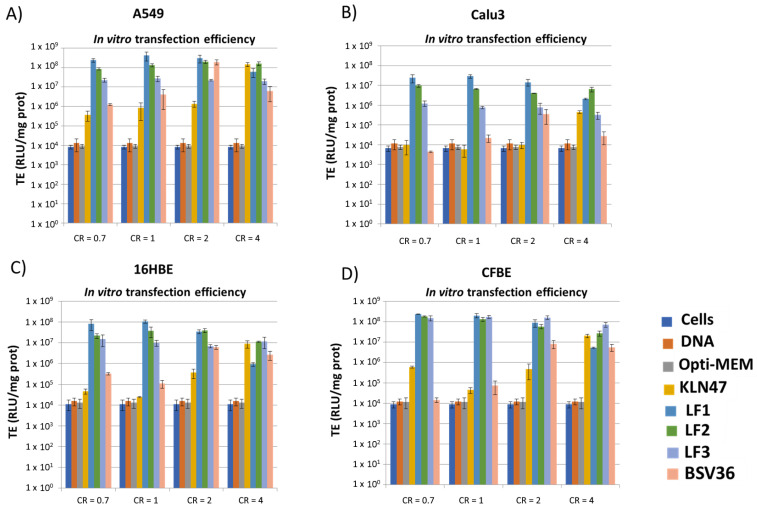
In vitro transfection efficacies (RLU/mg of protein) as a function of the charge ratios (CR from 0.7 to 4) in four cell lines: (**A**) A549; (**B**) Calu-3; (**C**) 16HBE; (**D**) CFBE.

This procedure was similar to a previously reported protocol (Le Corre et al., 2014 [37]). Briefly, cells were grown in EMEM (16HBE and CFBE) or DMEM (A549, Calu-3), both supplemented with 10% fetal bovine serum, 1% antibiotic and 1% L-glutamine at 37 °C in a humidified atmosphere containing 5% CO_2_. Cells were seeded onto a 96-well plate at a density of 20,000 to 40,000 cells per well for 24 h before transfection and incubated at 37 °C overnight. Complexes (including 0.25 µg of plasmid/well) were added dropwise to each well; the reference compounds **BSV36** and **KLN47** were used as positive transfection controls, whereas naked DNA was used as a negative control. After 24 h, the culture medium was removed and the cells were lysed with Passive Lysis Buffer (Promega) in order to be assayed for luciferase expression using a chemoluminescent assay (Luciferase Assay System, Promega). The total protein content of the cell lysate was determined using the BC assay kit (Uptima). Data are expressed as relative light units (RLU) per milligram of total protein (mean ± SD with *n* = 3), as depicted in Figure 4.

### 2.7. Cell Viability

**Figure 5:**
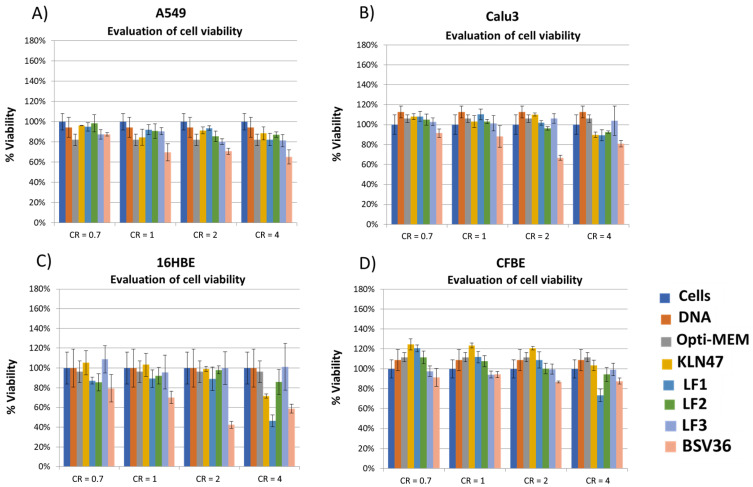
Cell viability as a function of the charge ratio (CR = 0.7 to 4). (**A**) A549; (**B**) Calu-3; (**C**) 16HBE; (**D**) CFBE.

This procedure was similar to a previously reported protocol (Delbeke et al., 2016 [38]). Briefly, the ATP content, which reflects the number of living cells (transfected or not) in culture, was determined using the ViaLight kit (Lonza, Basel, Switzerland) 24 h after transfection. This assay was used as recommended by the manufacturer. The results (Figure 5) were expressed as percentages relative to the viability of untransfected cells, which was used as the reference (100% cell viability).

## 3. Results and Discussion

The synthesis of the new cationic lipids is depicted in Figure 2. Regarding the choice of the lipid chains, we selected oleyl chains or the branched lipid chain 2-decyl-tetradecanyl because they proved their efficacy when incorporated in the structure of other cationic amphiphiles [23,35]. Previous publications have indeed observed that oleyl chains increase the fluidity of self-assemblies thanks to the presence of their carbon–carbon double bond possessing a Z-configuration. This structural feature introduces a kink in the lipid chain that disturbs the lipid packing and increases fluidity, as previously reported [39]. In the case of branched lipid chains, the ramification results in a larger volume of hydrophobic chains that contribute to the formation of a cone-shaped amphiphilic structure that favors non-lamellar self-assemblies and produces efficient gene carriers [23]. Their synthesis starts with a transesterification reaction involving a lipid alcohol and diphenylphosphite. This reaction can be achieved on large scale (10 g) and with high yields (>95%) in a Kugelrohr apparatus. In these conditions, phenol was removed from the products **1a–b** (Figure 2) by sublimation. Then, these phosphites were used for the preparation of the methylphosphonodithioformiates **2a–b** by adapting the method of Grisley [40]. The presence of the lipid chains reduced the reactivity of the phosphite. Accordingly, an efficient deprotonation required heating of the phosphite in the presence of sodium hydride in dry THF (reflux). Then, there was the addition of carbon disulfide at a low temperature, in order to avoid a desulfuration reaction [41], and finally, the methylation step produced the expected phosphonodithioformiates **2a–b** at modest yields (20%). This low yield was accounted for by the formation of side products (visible by ^31^P NMR) that imposed the loss of some of the expected product that was eluted in the same fractions as the by-products. Then, the phosphonodithioester–amine coupling (PAC) reaction was achieved by mixing **2a–b** with *N,N*-dimethylethylenediamine. The color of the reaction changed immediately from deep purple to yellow when *N,N*-dimethylethylenediamine was added—indicating a fast reaction, as previously reported for this type of reaction [32]. The resulting phosphonothioamides **3a–b** were methylated to produce the cationic amphiphilic compounds **4a–b**. Then, **4a–b** were formulated for the preparation of liposomal solutions. We observed that their formulation alone by either hydration of a lipid film or ethanol-injection methods produced very large aggregates with very high values on the PolyDispersity Index (PDI). In consequence, we associated **4a–b** with DOPE (1/1 molar ratio with either **4a** or **4b**) or DOPE/cholesterol as co-lipids in order to facilitate the formulation step. The resulting formulations were identified as LF1, LF2 and LF3 (Table 1). It must be noted that the incorporation of cholesterol (LF2 vs. LF1) reduced the transfection efficacy (as discussed later); as a consequence, the addition of cholesterol into LF3 was not considered in this study. These formulations were characterized by dynamic light scattering (DLS) to determine their size and zeta potential. The size of the aggregates (Table 2) was distributed over a quite narrow range of values (from 194 to 229 nm), and the zeta potentials were clearly positive—as expected for formulations including cationic amphiphilic compounds (+57 to +79 mV). After storing these formulations for 5 months, new analyses (Table S1) indicated that these formulations were still suitable for use as transfection agents (size < 200 nm; positive zeta potentials). The sizes and zeta potentials of the lipoplexes were also recorded (Table S2). It was observed that the size of the lipoplexes (depending on the charge ratios) were either similar or slightly smaller. For the zeta potential, positive values were observed at CR = 2 or 4 for all the formulations except for LF3. Indeed, for LF3, a slightly negative zeta potential (−13 to −10 mV) was observed at the different charge ratios. This negative charge could be due to a different type of supramolecular assembly. Branched lipid chains are indeed prone to adopting inverted hexagonal assemblies [23]. The complexation properties of these formulations were evaluated using gel electrophoresis of the lipoplexes, prepared in water at different charge ratios (from 0.7 to 4; Figure 3). It appeared that LF1 fully compacted pDNA at CR = 2, whereas LF2 required CR = 4 to produce full complexation. LF3 was less efficient at compacting pDNA, since even at CR 4 pDNA was only partly compacted. This behavior could result from non-lamellar packing [23]. These formulations were then tested as gene carriers for the transfection of four cell lines (A549, Calu3, CFBE, 16HBE), and with pGM144 plasmid [42] used as a gene reporter (a plasmid encoding luciferase). For these experiments, we used **BSV36** and **KLN47** as references because we have previously reported their transfection efficacies [35]. **KLN47** has also been previously compared to other commercial kits of transfection [43]. The new cationic amphiphiles **4a** and **4b** formulated with DOPE (LF1 and LF3; Table 1), or with DOPE and cholesterol (LF2; Table 1), were very efficient most of the time at transfecting all the cell lines, and were therefore good candidates compared to other nucleic acid carriers [44] for use in further developmental studies. The most remarkable results came from the formulation LF1, which proved to be very efficient at transfecting the four cell lines tested at relatively low charge ratios (CRs = 0.7 to 2; Figure 4). The effects of the transfection conditions on cell viability were studied and the results are depicted in Figure 5. Overall, the formulations exhibited low toxicity at CR = 0.7, 1 and 2. Some toxicity was observed at CR 4 for **BSV36** in some cell lines (A549, Calu3, 16HBE). However, at the CRs efficient for transfection (CR = 0.7, 1 or 2), the most efficient formulations—LF1 and LF2—were not toxic under the tested conditions. These results confirm the interest of the formulations LF1 and LF2, which include cationic amphiphilic compounds that are prepared using the PAC reaction in order to include in their molecular structures a phosphonothioamide moiety.

## 4. Conclusions

For the first time, cationic amphiphilic compounds have been prepared using a phosphonodithioester–amine coupling (PAC) reaction. The final cationic amphiphilic compounds **4a–b** were isolated in a four-step synthesis scheme in sufficient quantities for their evaluation as plasmid DNA carriers. The interest of the PAC reaction comes from its rapidity and from its efficacy without the requirement of any catalyst. These cationic amphiphilic compounds were formulated in the presence of DOPE or DOPE/cholesterol to produce well-defined liposomal solutions (size close to 200 nm). These two cationic amphiphilic compounds included in their structure either oleyl chains or branched lipid chains that had previously proved their efficacy when incorporated into other type of cationic amphiphilic structures. The formulations, including **4a–b,** feature different capacities for compacting pDNA. Among these formulations, the one featuring the highest capacity to compact pDNA (LF1) was also the most efficient formulation for the transfection of four cell lines (A549, Calu3, CFBE, 16HBE). Compound **4a,** formulated with either DOPE or DOPE/cholesterol (LF1 or LF2), was the most efficient formulation for transfecting the four cell lines. The most remarkable results were that LF1 and LF2 were efficient at low charge ratios (at charge ratios where the complexation of pDNA was incomplete), suggesting that such partial complexation could be helpful for high in vitro transfection efficacies—likely by facilitating the release of the plasmid from the endosome to the cytosol. The side effects induced by these formulations were evaluated by determining cell viability. The most efficient formulations, LF1 and LF2, exhibited no toxicity at the charge ratio where they were the most efficient (CR = 0.7, 1 or 2). The influence of the lipid chain on the transfection efficacy was in favor of the oleyl chains (LF1 and LF2). With the cationic amphiphile-featuring branched lipid chain (LF3), lower transfection efficiencies were observed—except for CFBE cell line. Altogether, the cationic amphiphilic **4a** compounds formulated with DOPE (LF1) produced very promising results in terms of transfection efficacies, coupled with the absence of toxic effects. The synthetic approach, based on the PAC reaction, opens up new possibilities for the modulation of both the polar head group and the hydrophobic domain.

## Figures and Tables

**Table 1:** Composition and method used to prepare the formulations LF1–3 and the reference formulations **BSV36** and **KLN47**.

Formulations	Composition	Ratio	Concentration	Method
BSV36	BSV36	1	1.5 mM	Lipid film hydration
KLN47	KLN47	1	1.5 mM	Lipid film hydration
LF1	**4a**: DOPE	1: 1	1.5 mM in **4a**	Lipid film hydration
LF2	**4a**: Chol: DOPE	1: 1: 1	1.5 mM in **4a**	Lipid film hydration
LF3	**4b**: DOPE	1: 1	1.5 mM in **4b**	Ethanol injection

**Table 2:** Sizes and zeta potentials of liposomal solutions.

Formulations	Size (nm)	PDI	Potential Zeta (mV)
BSV36	169 ± 9	0.24	58 ± 0.06
KLN47	126 ± 7	0.33	28 ± 1.16
LF1	227 ± 41	0.46	61 ± 1.7
LF2	194 ± 16	0.44	79 ± 0.6
LF3	229 ± 15	0.33	57 ± 0.8

## Supplementary Materials

The following are available online, in the supplementary materials part: Figures S1–S35 correspond to NMR and MS spectra (^1^H, ^31^P, ^13^C, MALDI TOF or ESIqTOF MS spectra) for the compounds 2–4/a–b, Table S1 “Sizes and zeta potentials of the liposomes at t = 0 and 5 months later.”, Table S2 “Sizes and zeta potentials of the lipoplexes at different charges ratios”, Table S3 “Fraction of complexed DNA and free DNA after complexation process with the cationic lipids determined by densitometry analysis on the electrophoresis gel”, Figure S36 “Proportion of complexed and free DNA after complexation process with the cationic lipids determined by densitometry analysis on the electrophoresis gel.”
