# Supplementary material for: Phosphonodithioester–Amine Coupling as a Key Reaction Step for the Design of Cationic Amphiphiles Used for Gene Delivery
Source: Molecules. 2021 Dec 11;26(24):7507. doi: 10.3390/molecules26247507 (PMC8707180; doi:10.3390/molecules26247507)
Supplement: Supplementary file 1 [file molecules-26-07507-s001.zip › molecules-1476714-supplementary.pdf]

Supporting materials

Phosphonodithioester-Amine Coupling as a key reaction to design cationic amphiphiles used for gene delivery.

Montassar Khalil[a], Alexis Hocquigny,[b] Mathieu Berchel,[a] Tristan Montier,[b] Paul-Alain Jaffrès[a]

Summary

1. Analysis (NMR, IR, Masse) 2
2. Stability of the liposomes 21
3. DLS and zeta potential of lipoplexes 22
4. Gel electrophoresis 23

1. Analysis (NMR, IR, Masse)

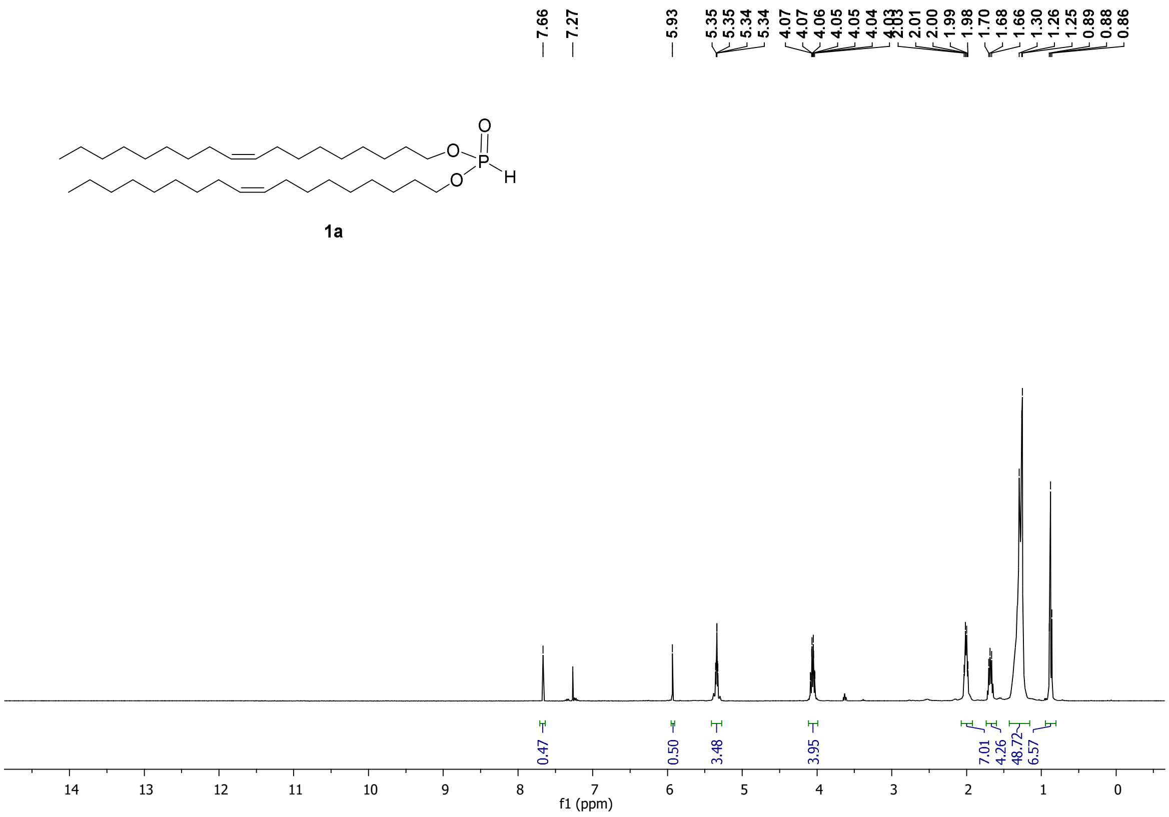

Figure S1. *¹H NMR ($CDCl_3$) of compound* ***1a***

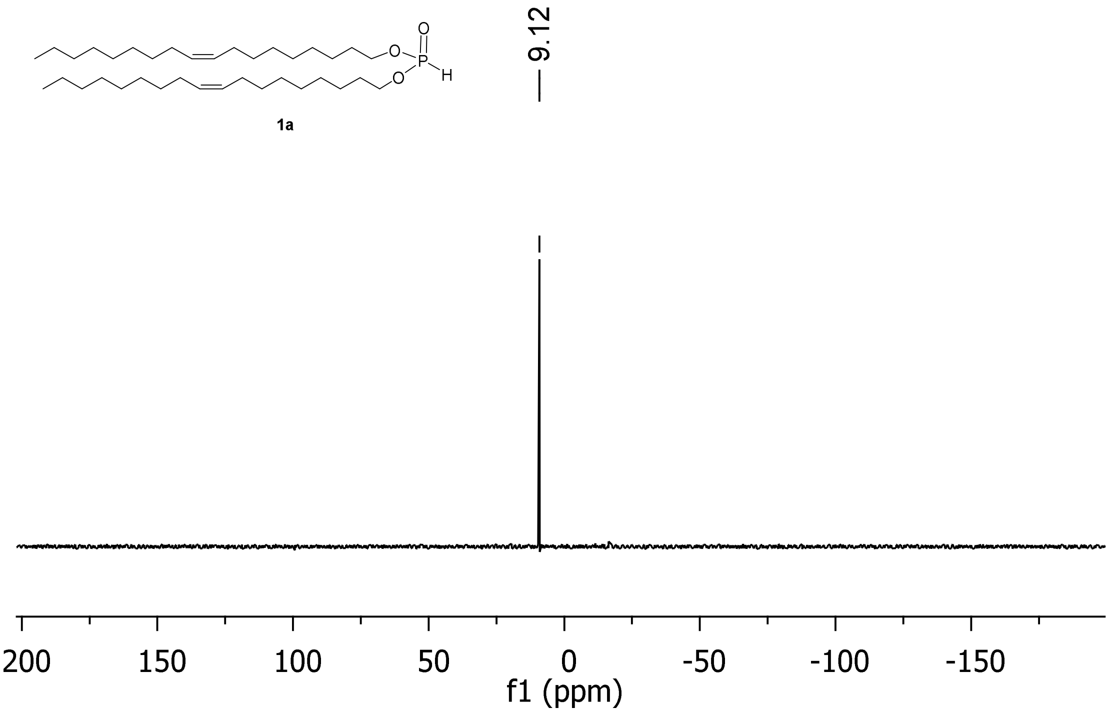

Figure S2. *³¹P {¹H} ($CDCl_3$) of compound* ***1a***

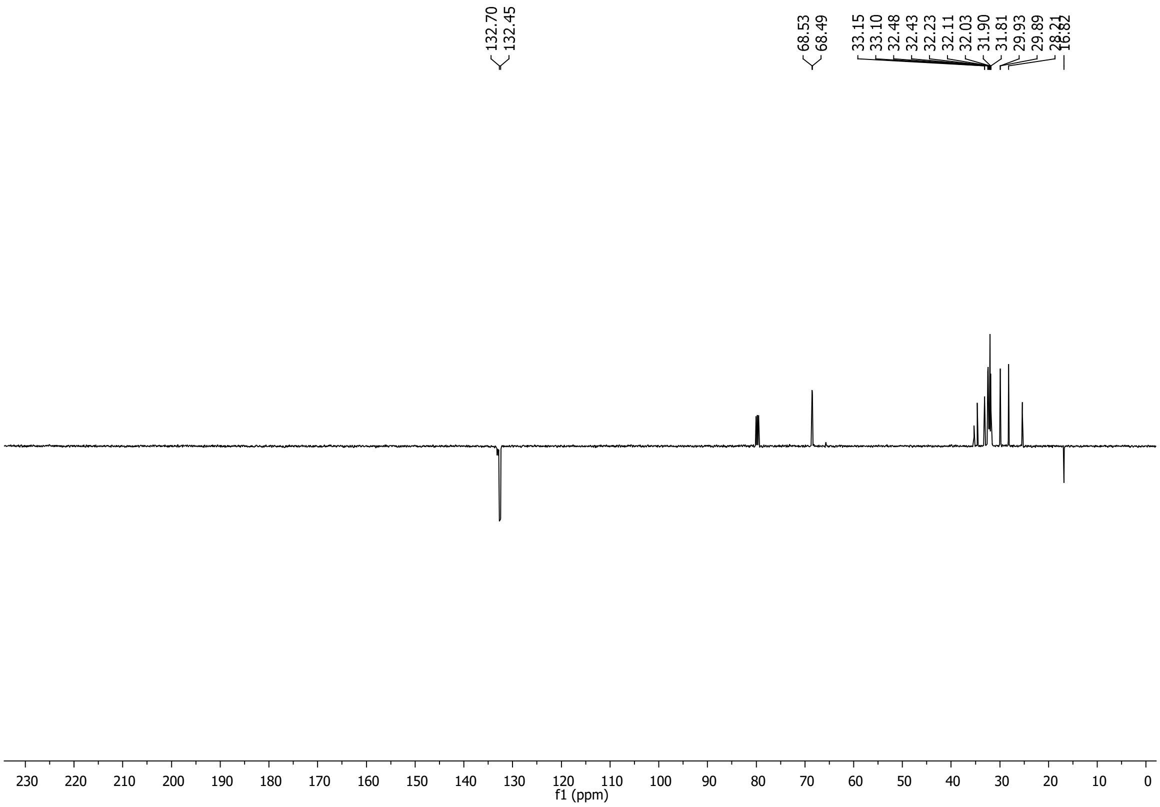

Figure S3. *$^{13}C\ \{^{1}H\}\ (CDCl_3)$ of compound* ***1a***

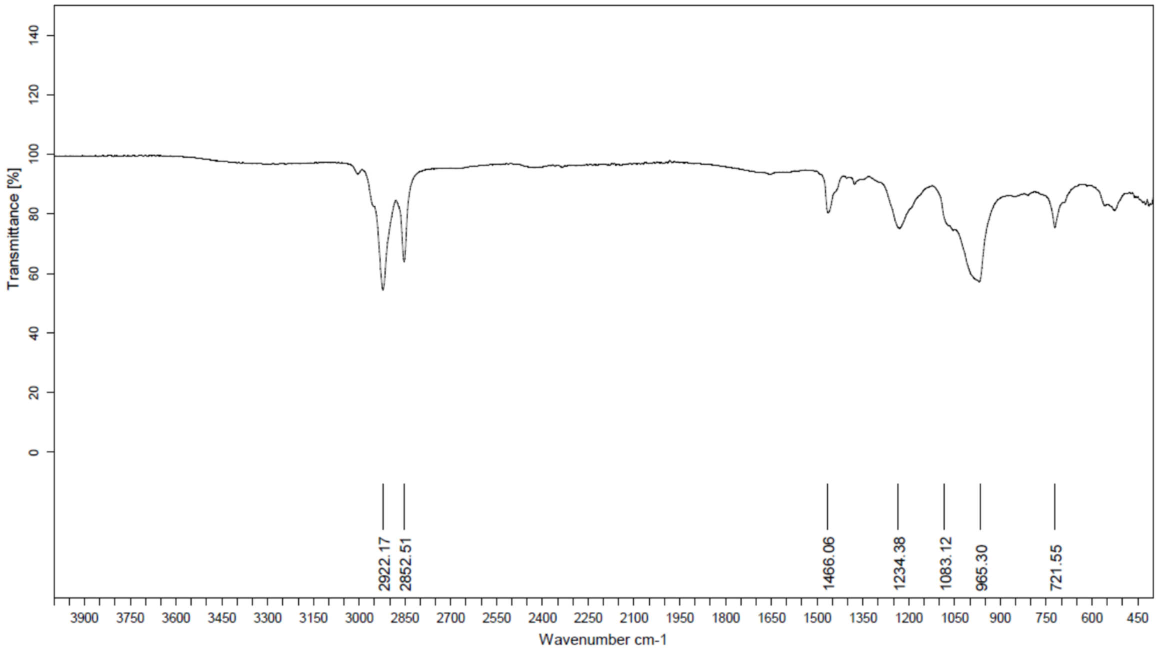

Figure S4. *IR (ATR) of compound* ***1a***

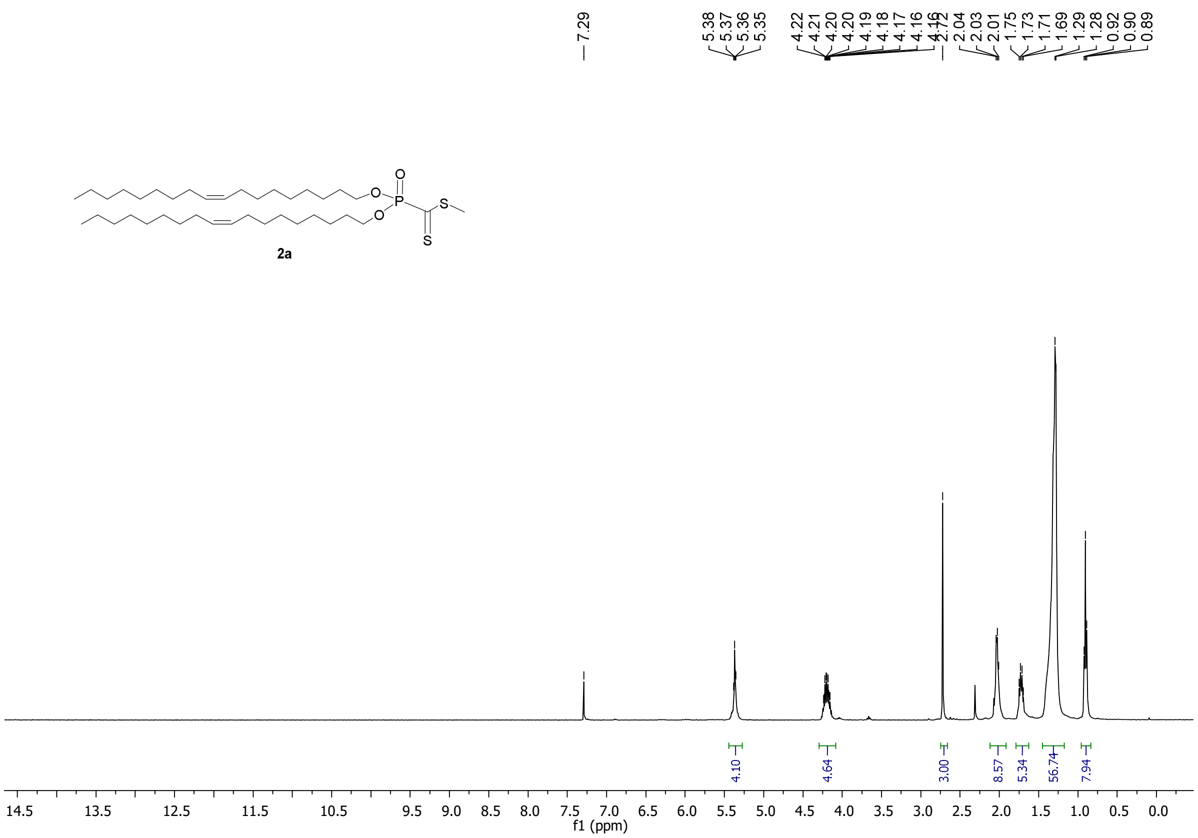

Figure S5**. ^{1}H NMR ($CDCl_3$) of compound **2a

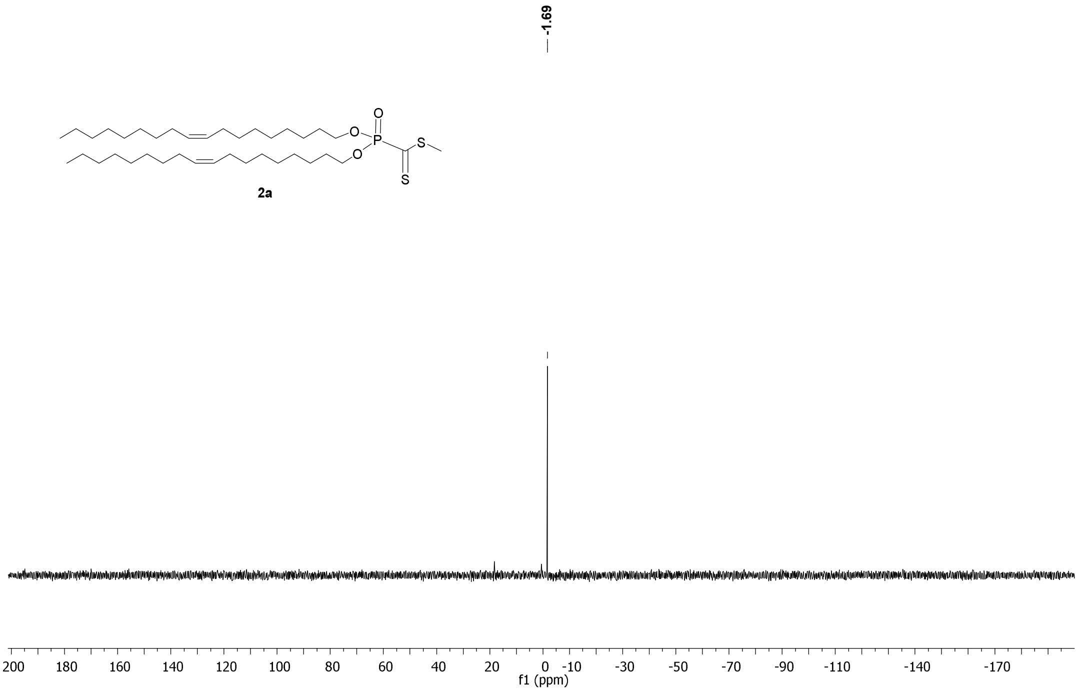

Figure S6**. ^{31}P {^{1}H} ($CDCl_3$) of compound **2a

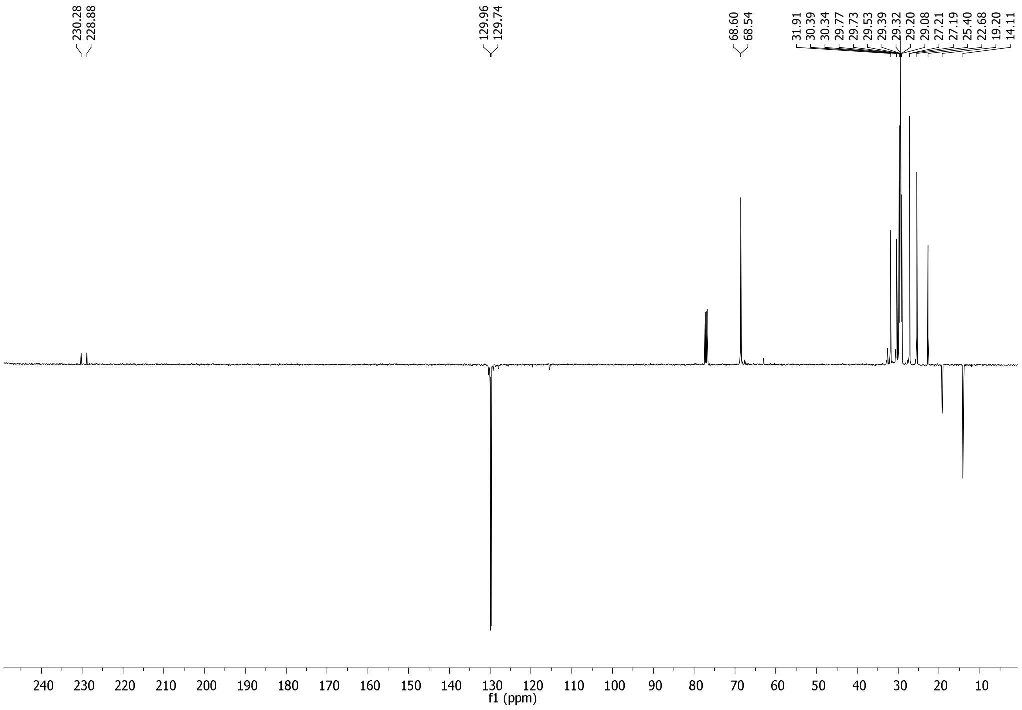

Figure S7. *^{13}C {^{1}H} ($CDCl_3$) of compound* ***2a***

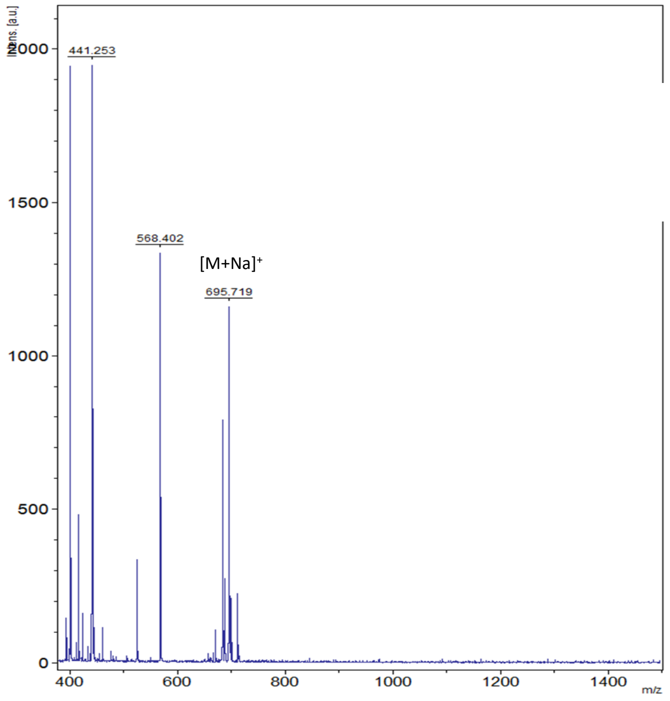

Figure S8. *MALDI-TOF (matrix : HCCA) of compound* ***2a***.

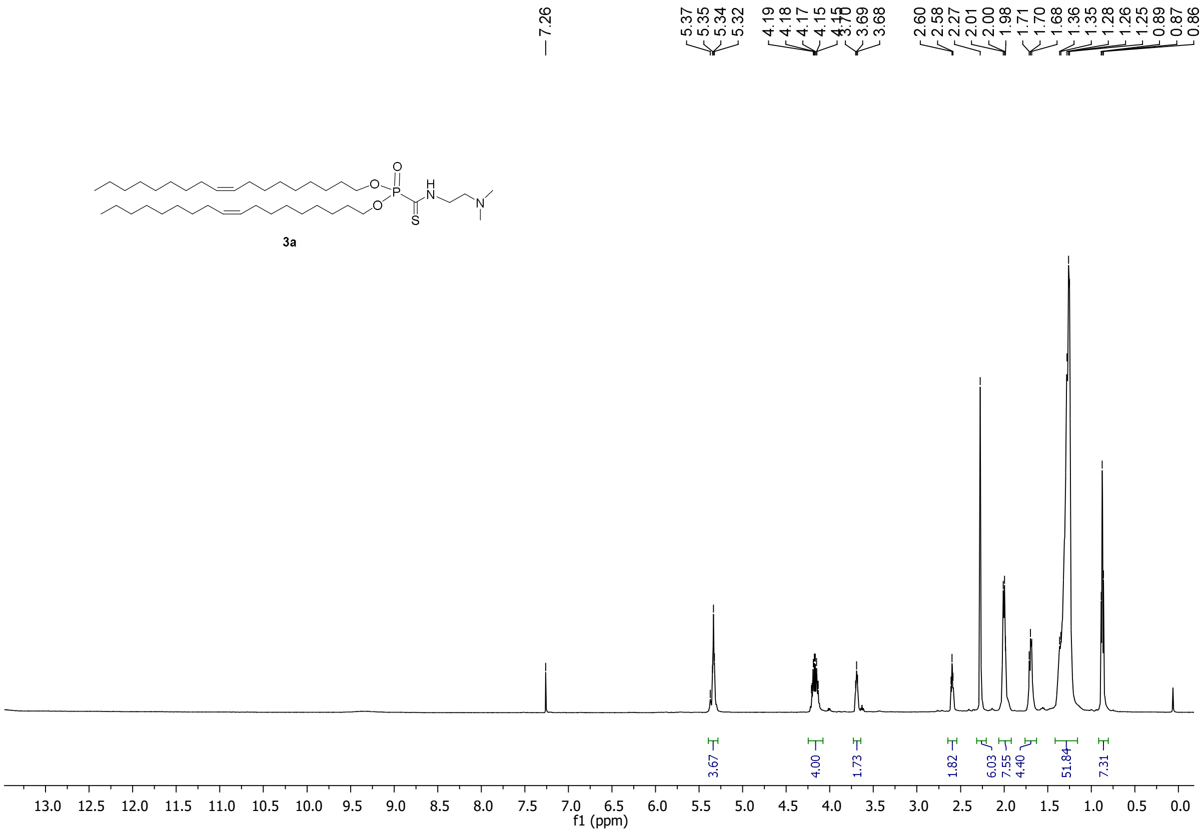

Figure S9. *^{1}H NMR (CDCl$_3$) of compound **3a***

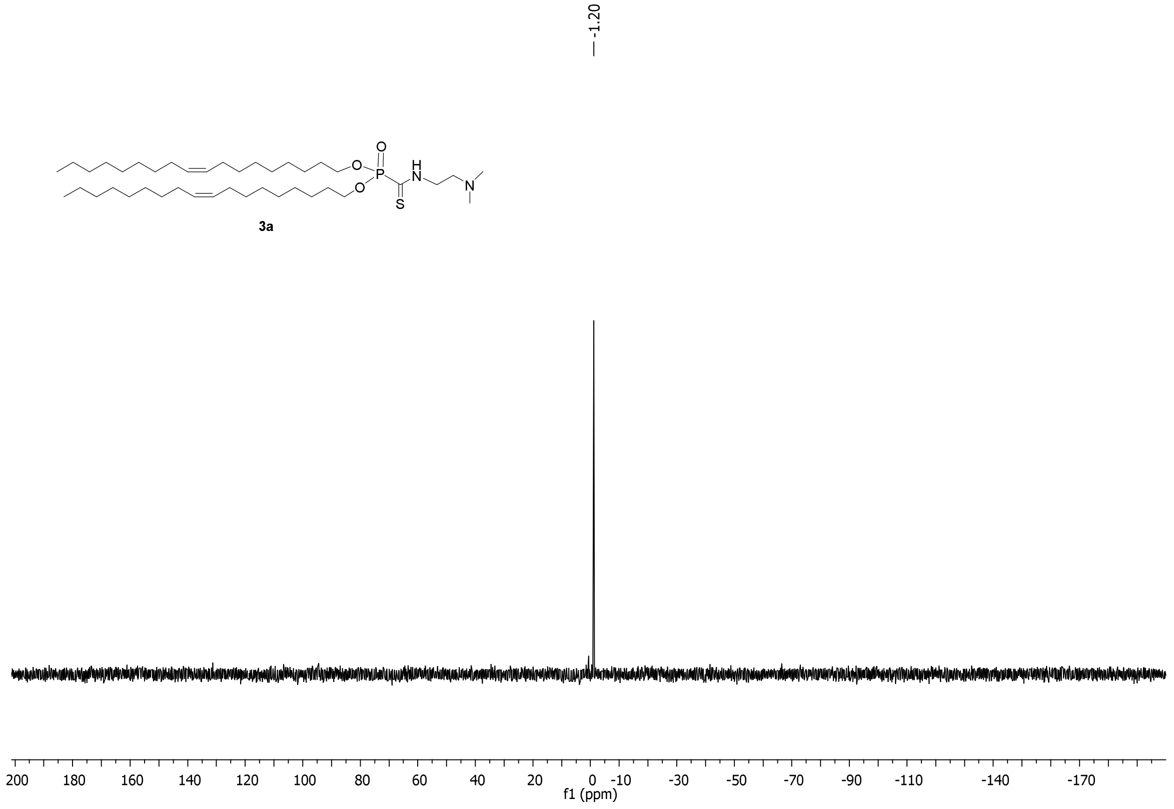

Figure S10. *[31]P {[1]H} (CDCl$_3$) of compound **3a***

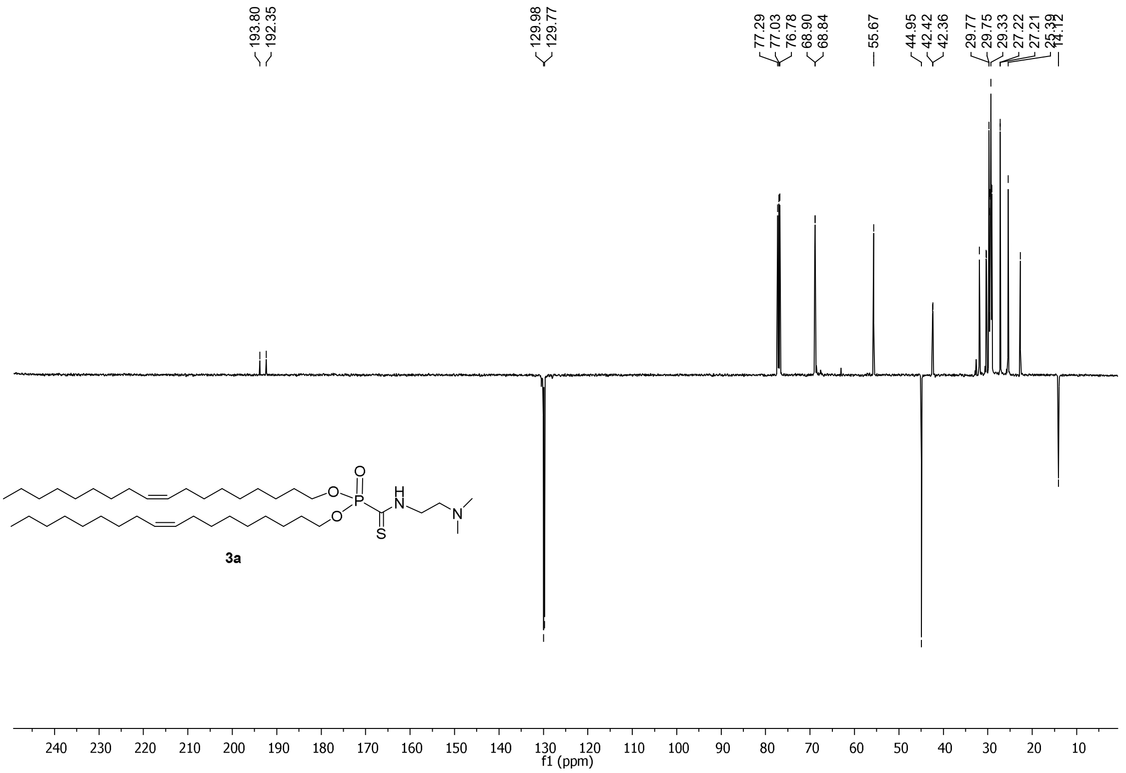

***Figure S11**. ^{13}C {^1H} ($CDCl_3$) of compound 3a*

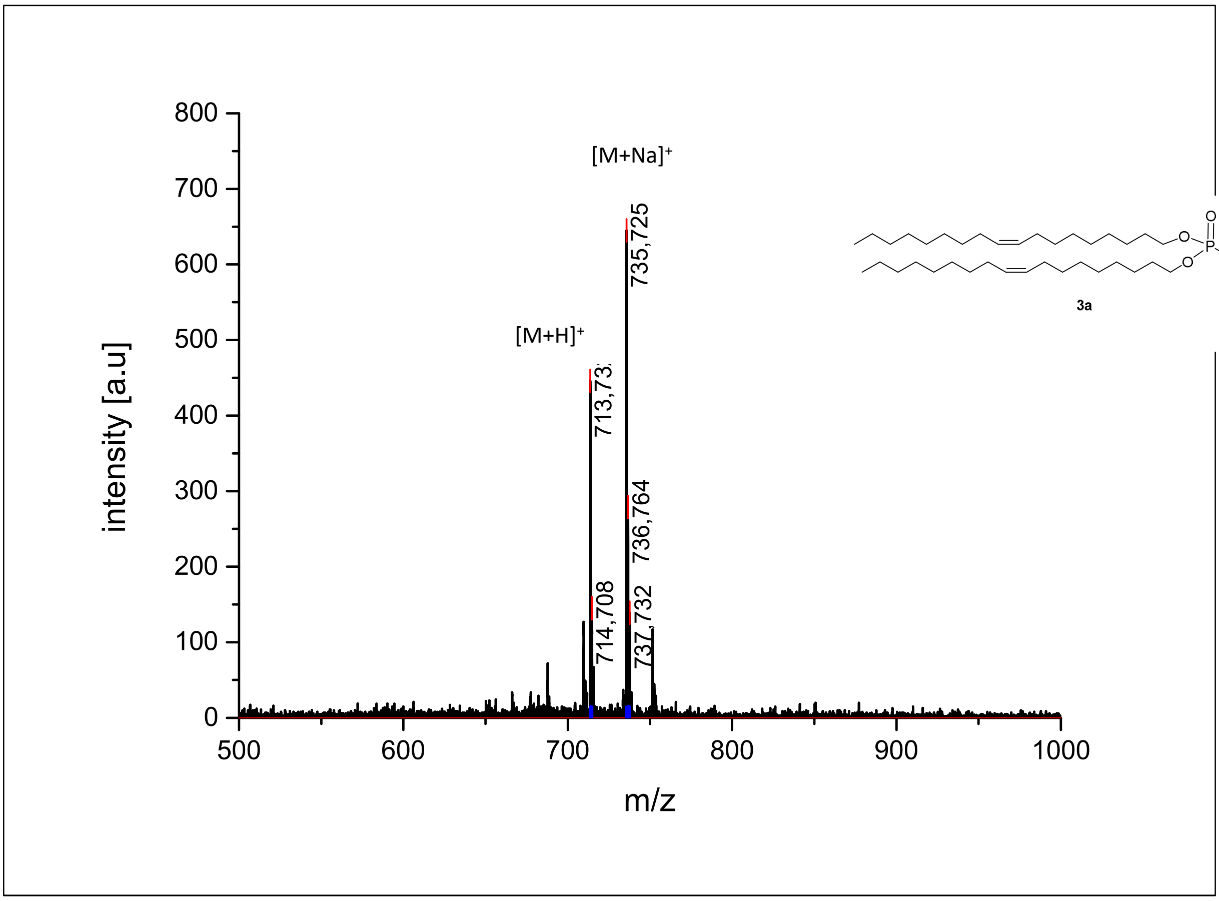

***Figure S12**. MALDI-TOF (matrix: HCCA) of compound **3a**.*

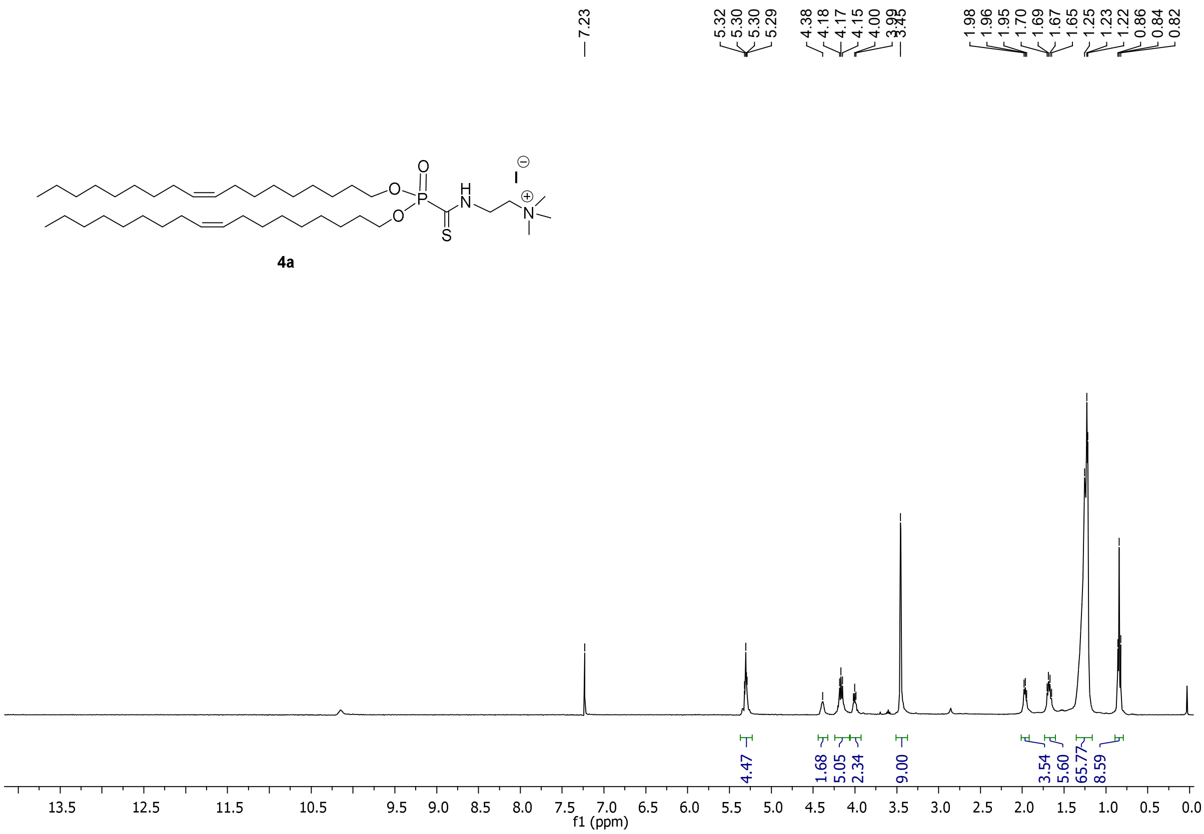

Figure S13. *^{1}H NMR ($CDCl_3$) of compound* ***4a***

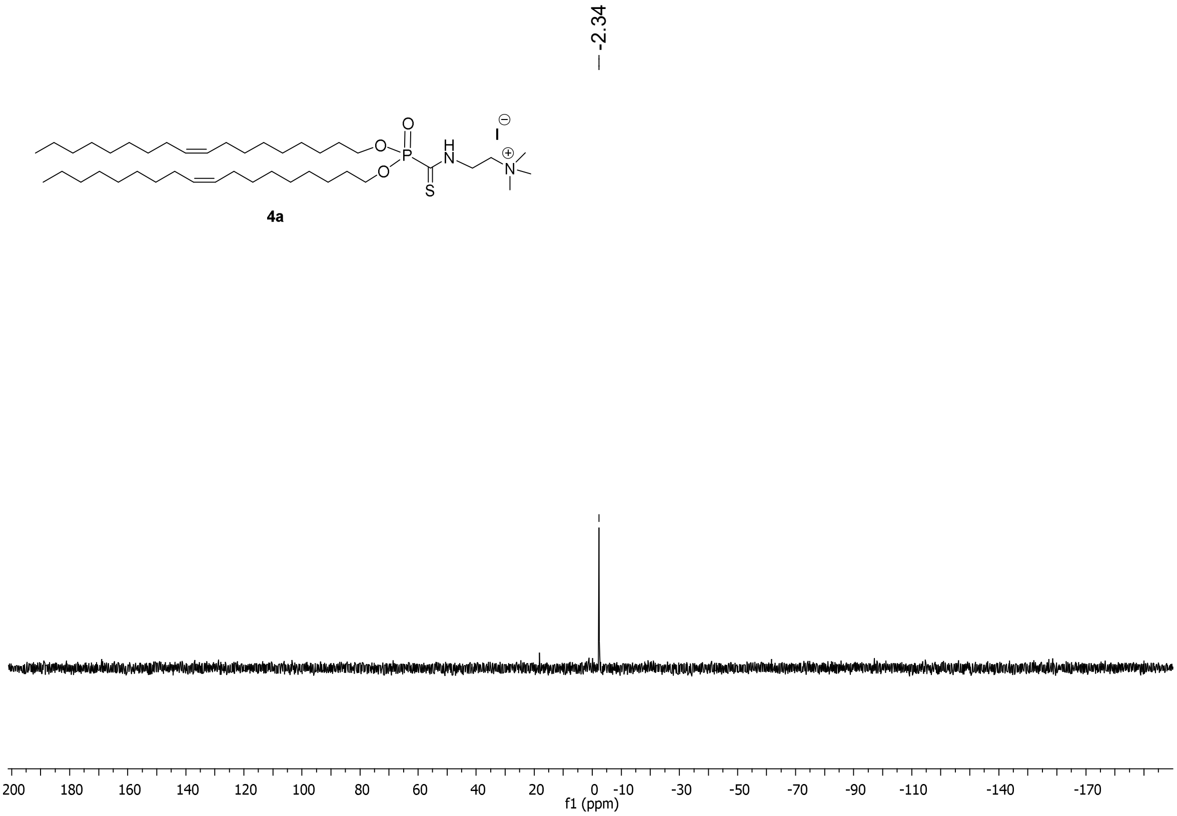

Figure S14. *^{31}P {^{1}H} ($CDCl_3$) of compound* ***4a***

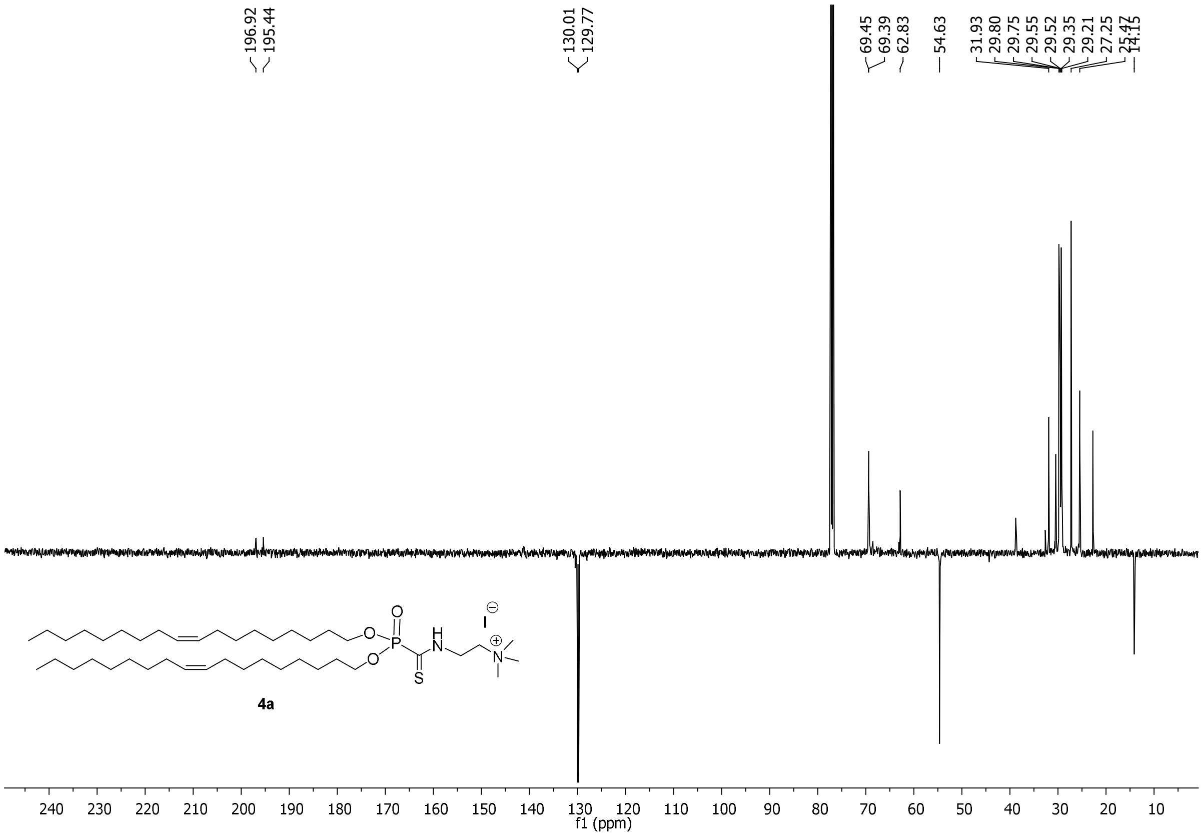

Figure S15. *$^{13}C\{^{1}H\}$ ($CDCl_3$) of compound **4a***

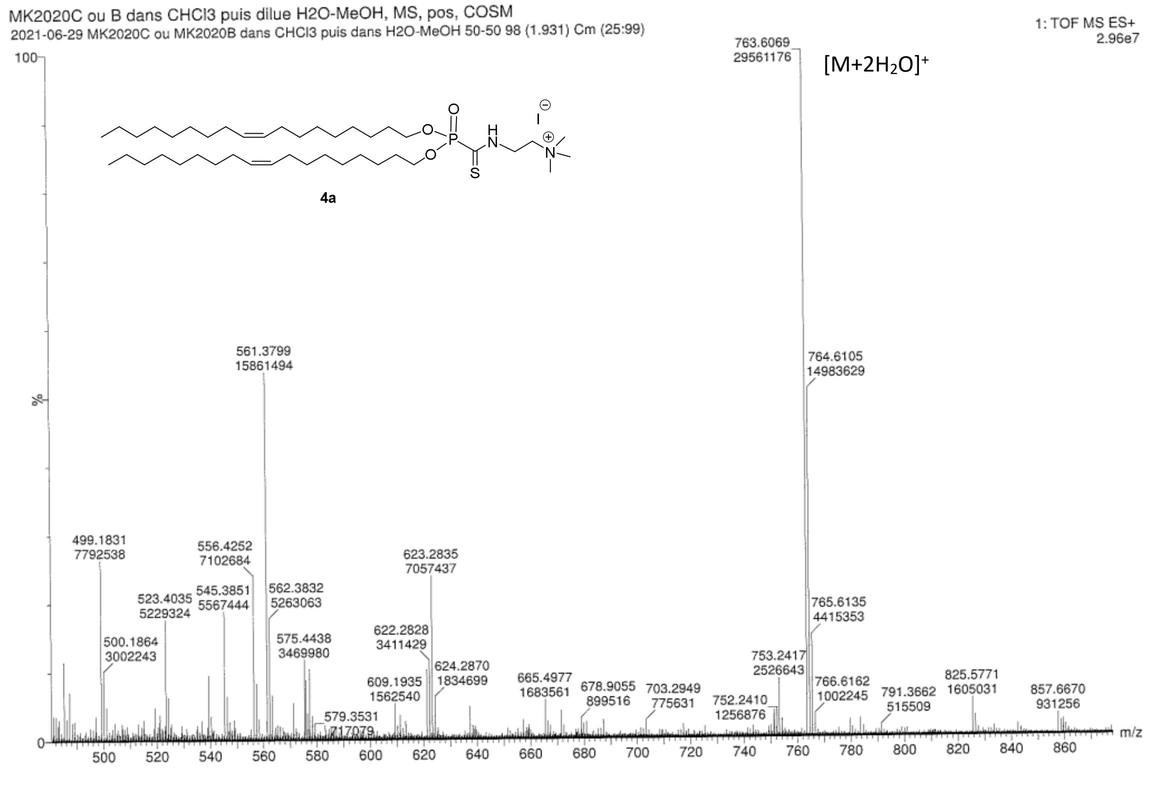

Figure S16. *ESI-qTOF MS of compound **4a***

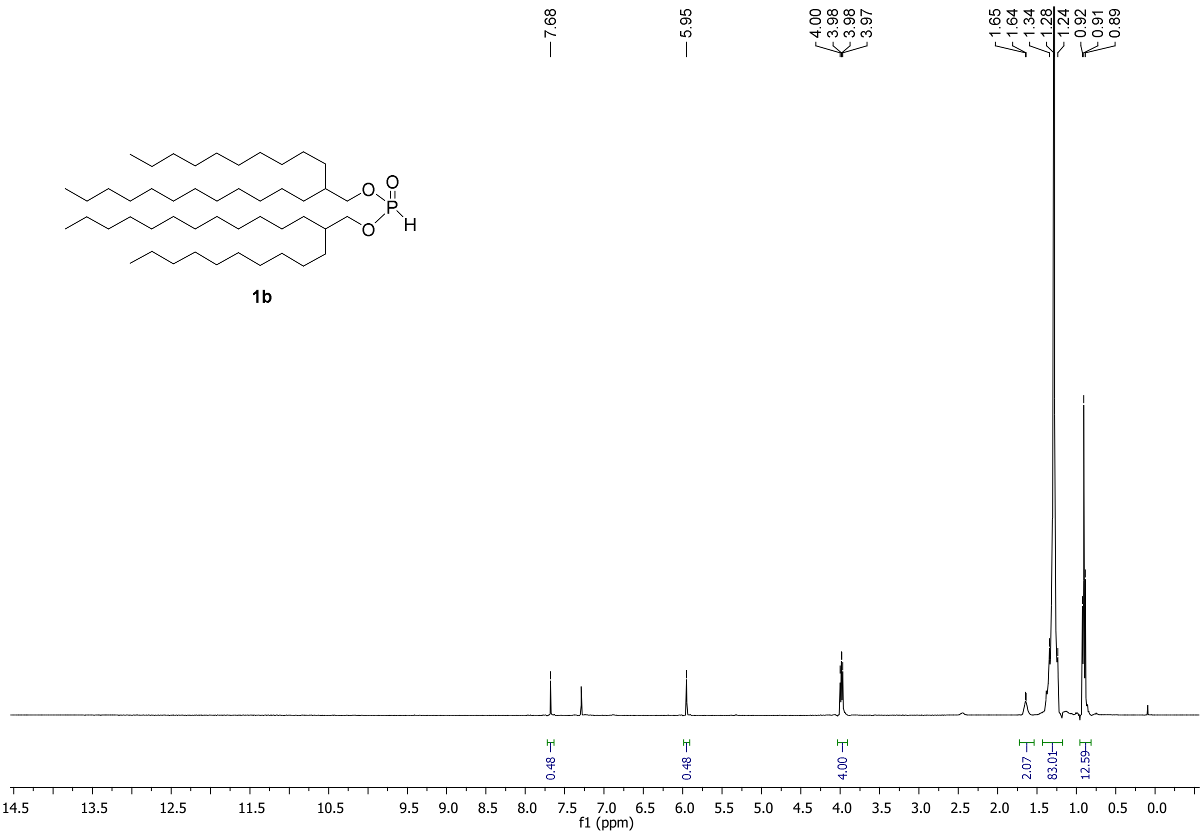

Figure S17. *^{1}H NMR ($CDCl_3$) of compound* ***1b***

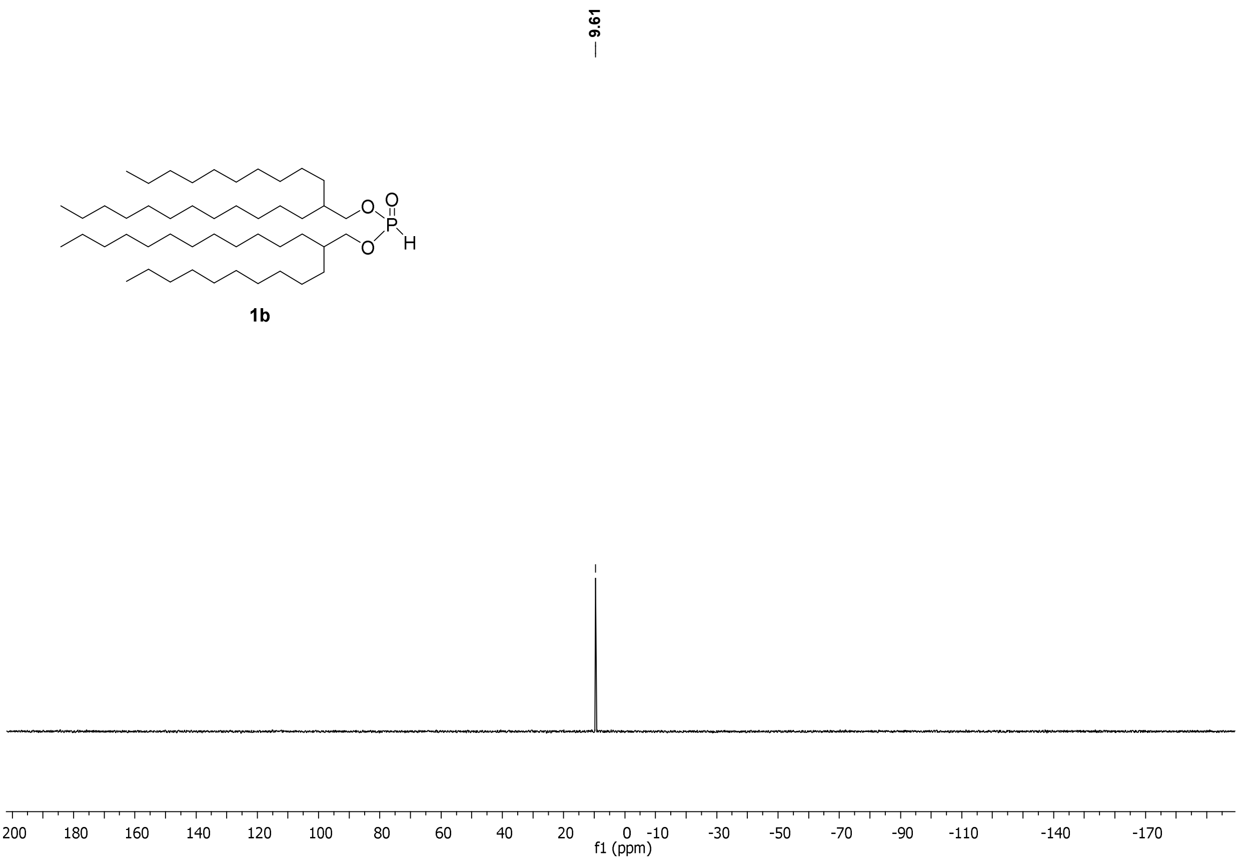

Figure S18. ^{31}P {^{1}H} ($CDCl_3$) *of compound **1b***

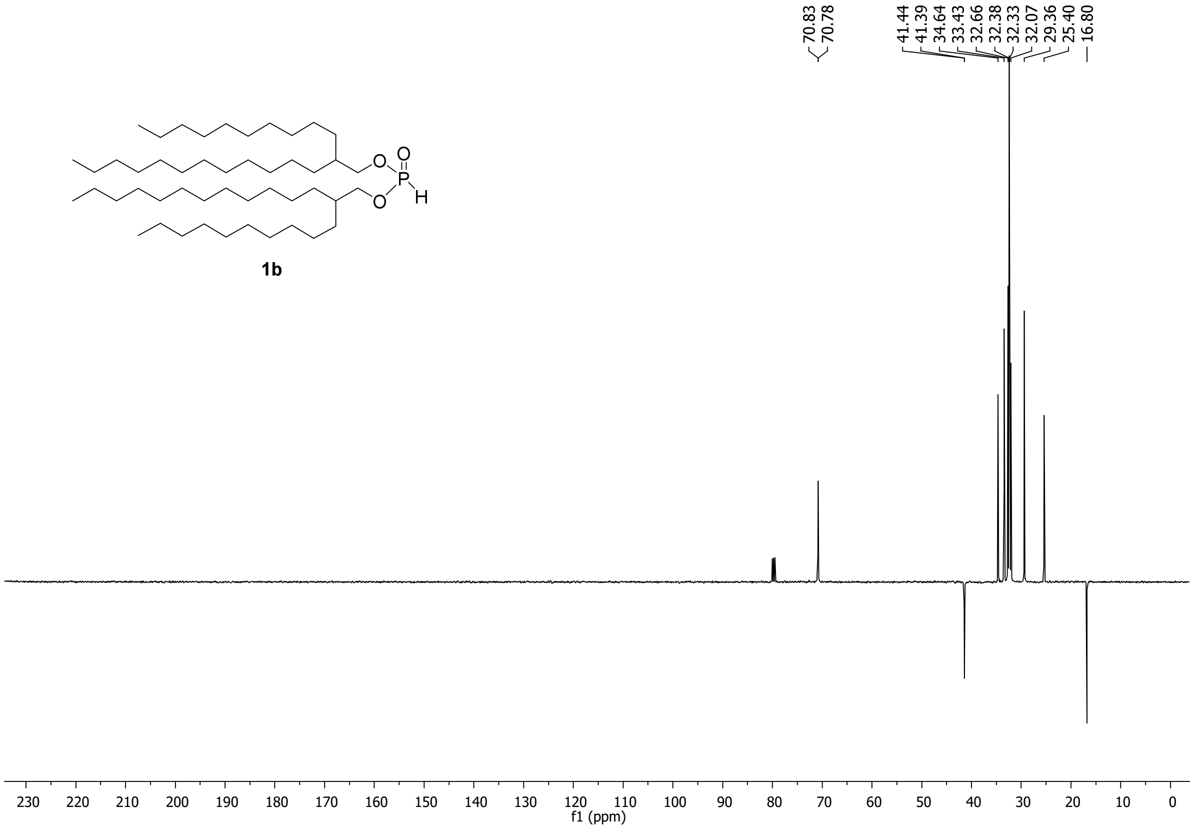

Figure S19. ^{13}C {^{1}H} ($CDCl_3$) *of compound **1b***

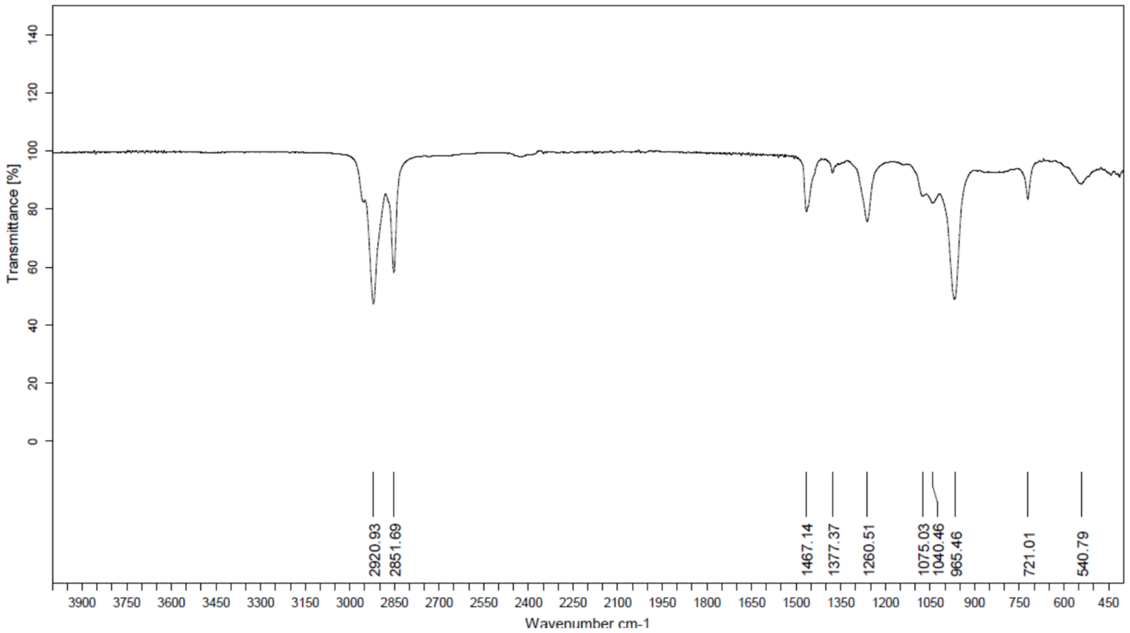

Figure S20**. IR (ATR) of compound **1b

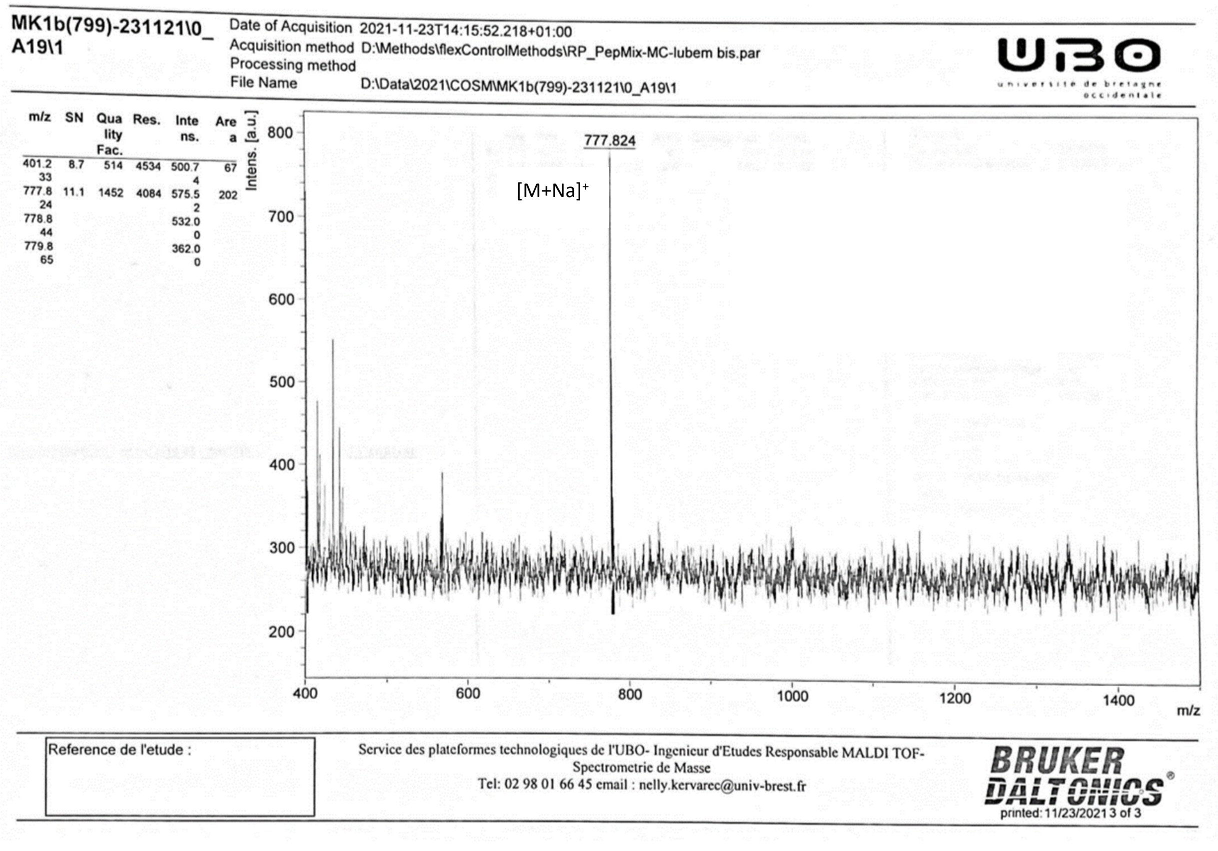

Figure S21**. MALDI-TOF (matrix HCCA) of compound **1b

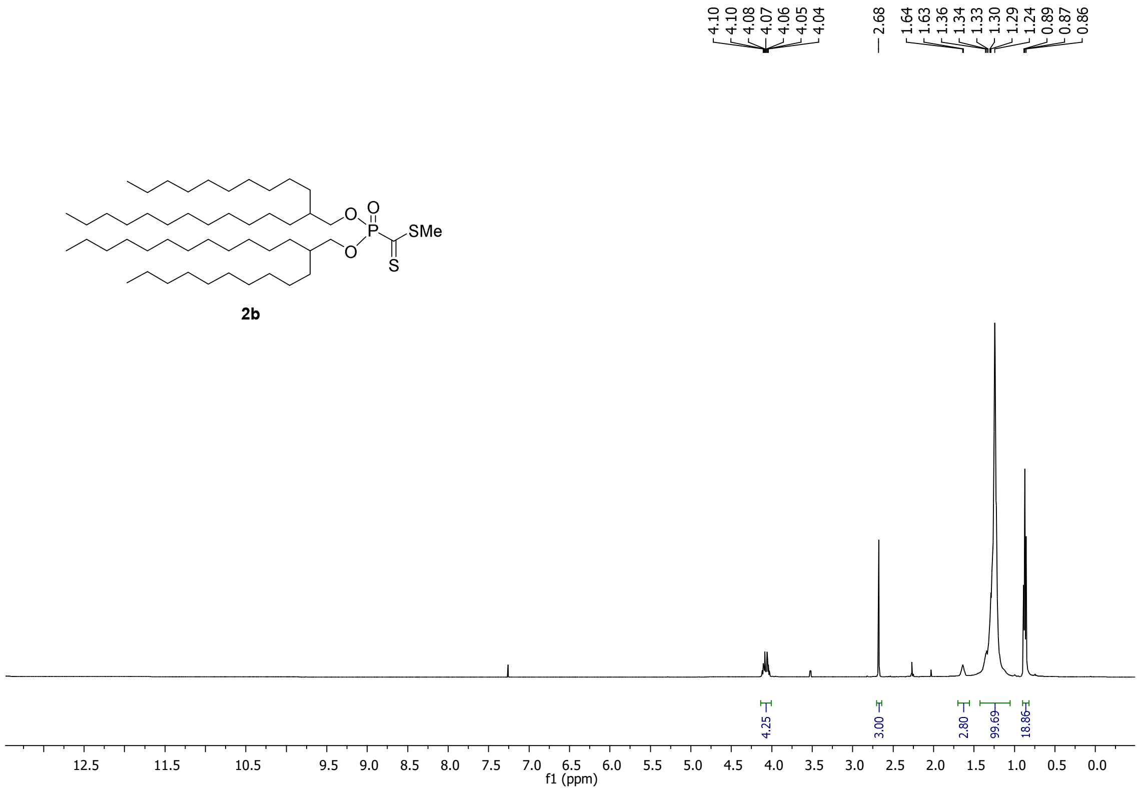

Figure S22. *^1H NMR ($CDCl_3$) of compound* ***2b***

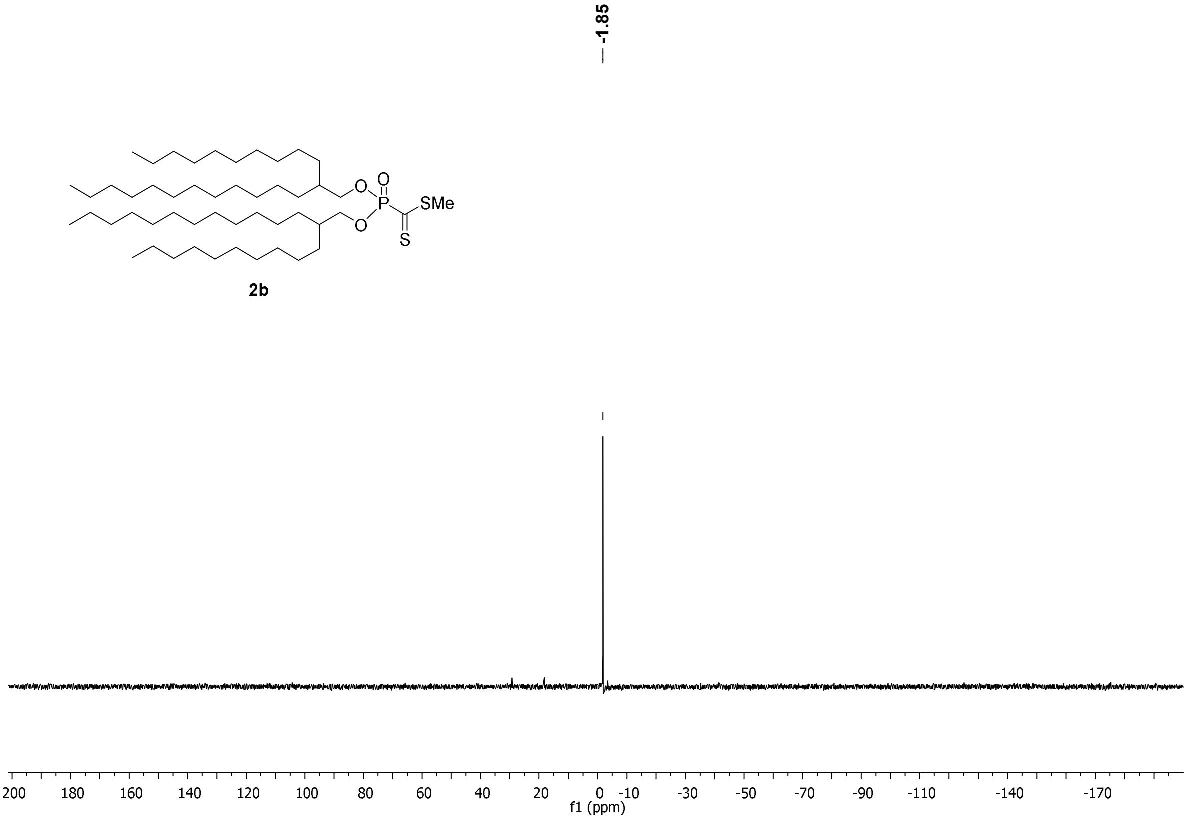

Figure S23. *^{31}P {^1H} ($CDCl_3$) of compound* ***2b***

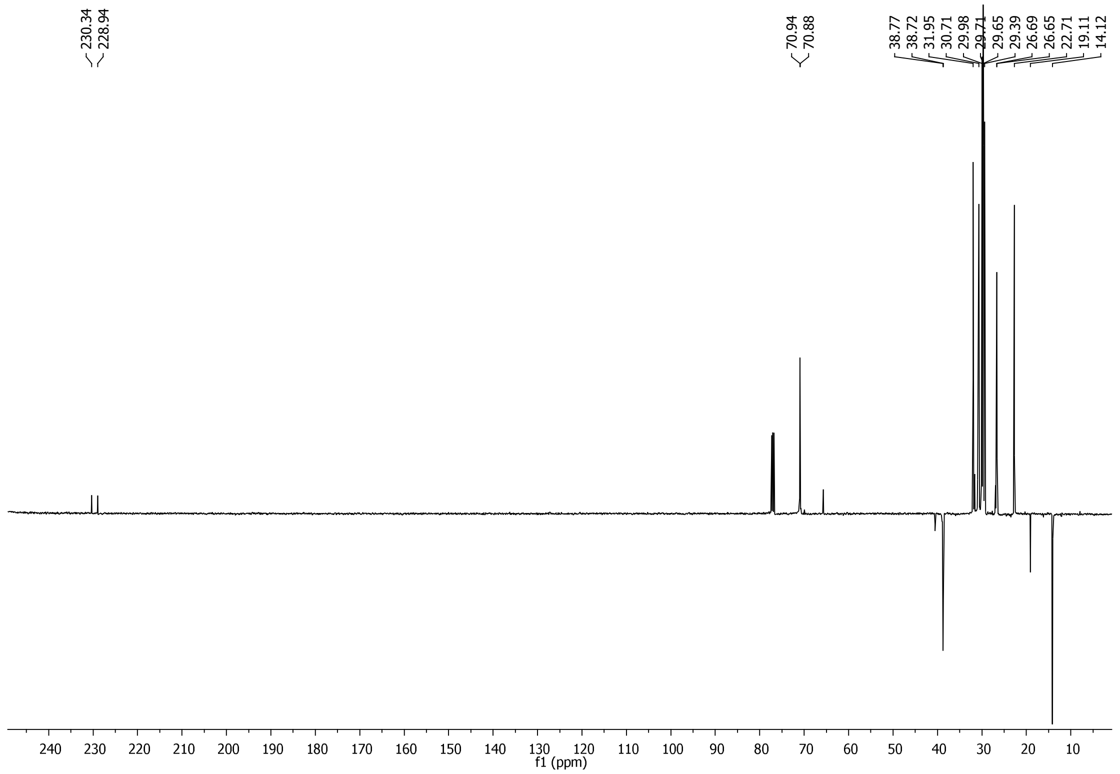

Figure S24. *^{13}C {^{1}H} ($CDCl_3$) of compound* ***2b***

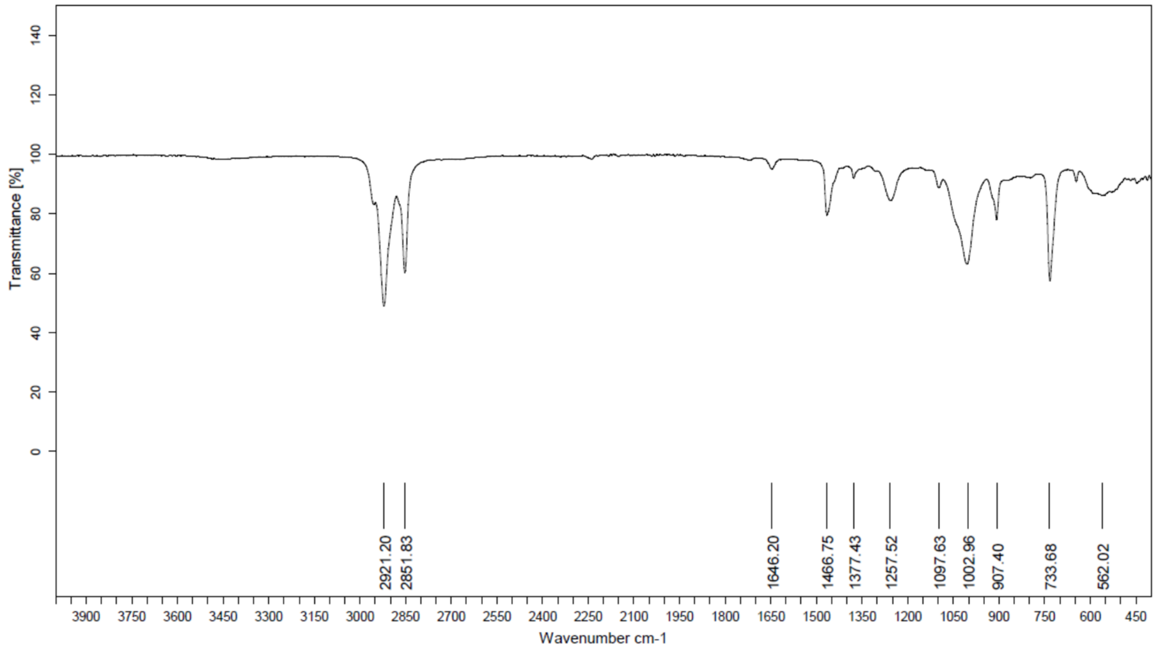

Figure S25. *IR (ATR) of compound* ***2b***

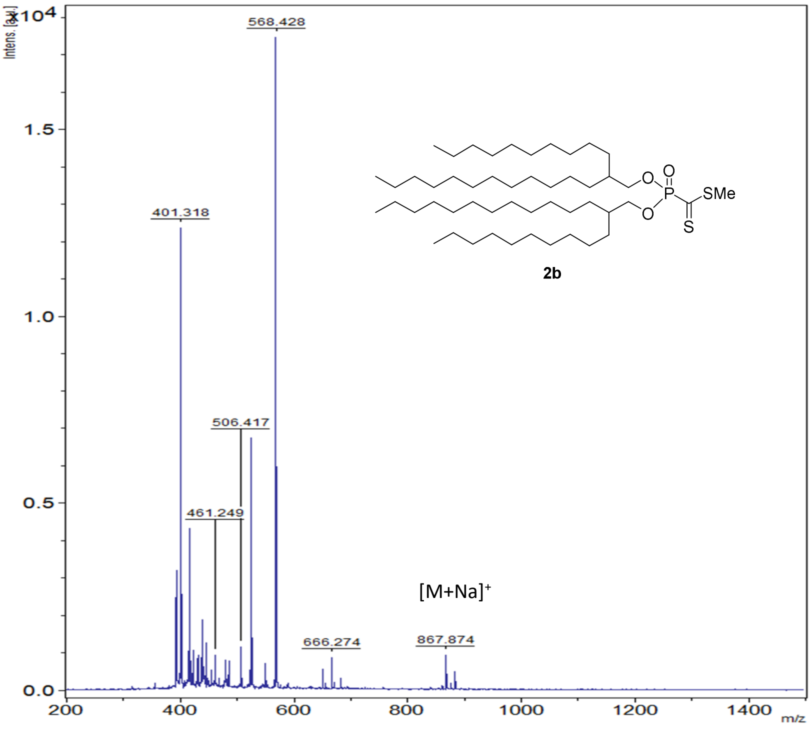

Figure S26**. MALDI-TOF (matrix: HCCA) of compound **2b

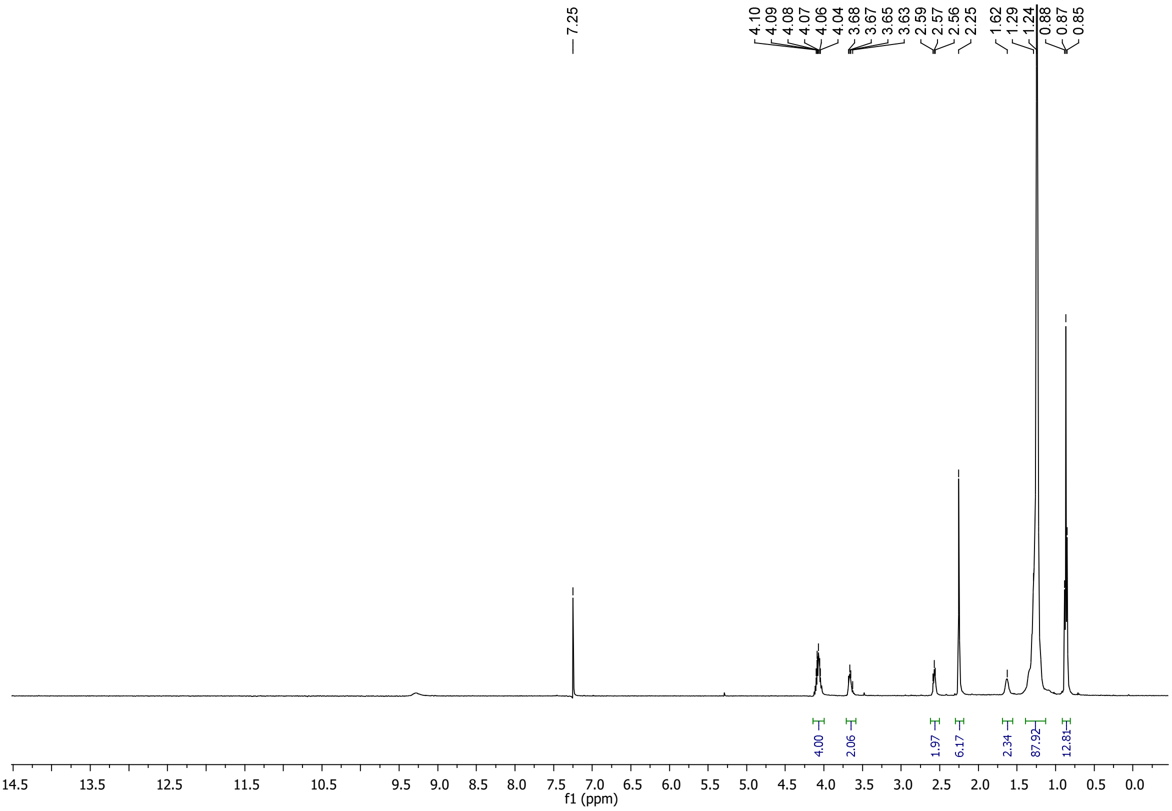

Figure S27**. ^{1}H NMR ($CDCl_3$) of compound **3b

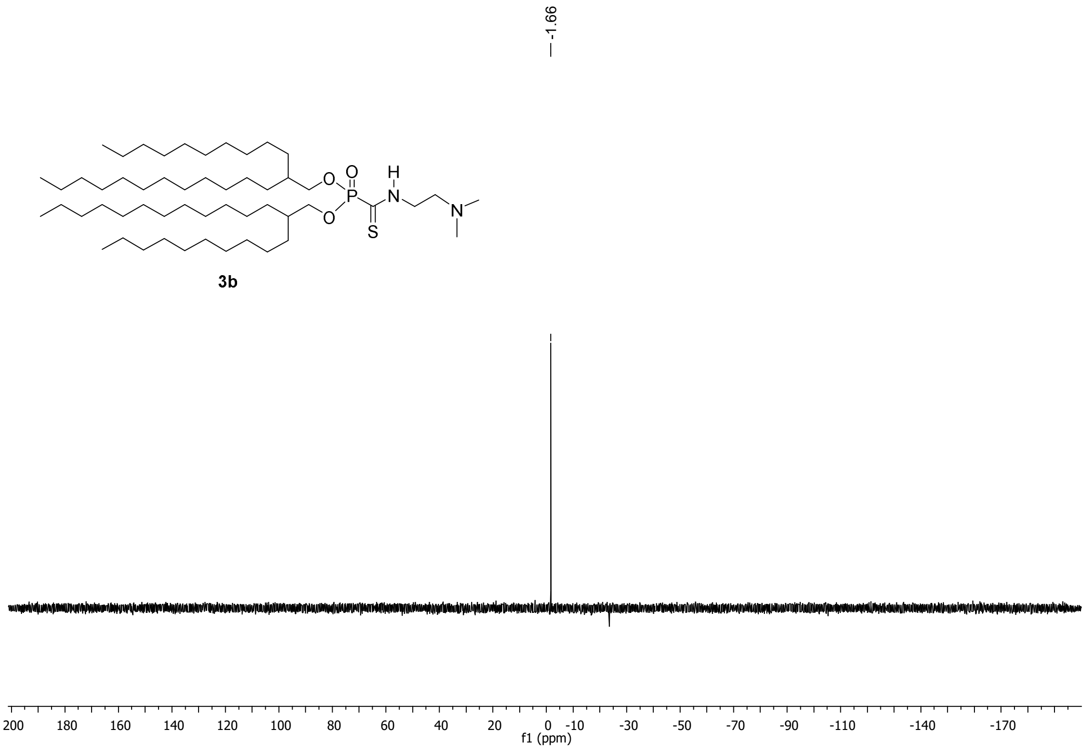

Figure S28. ^{31}P $\{^{1}H\}$ ($CDCl_3$) *of compound* ***3b***

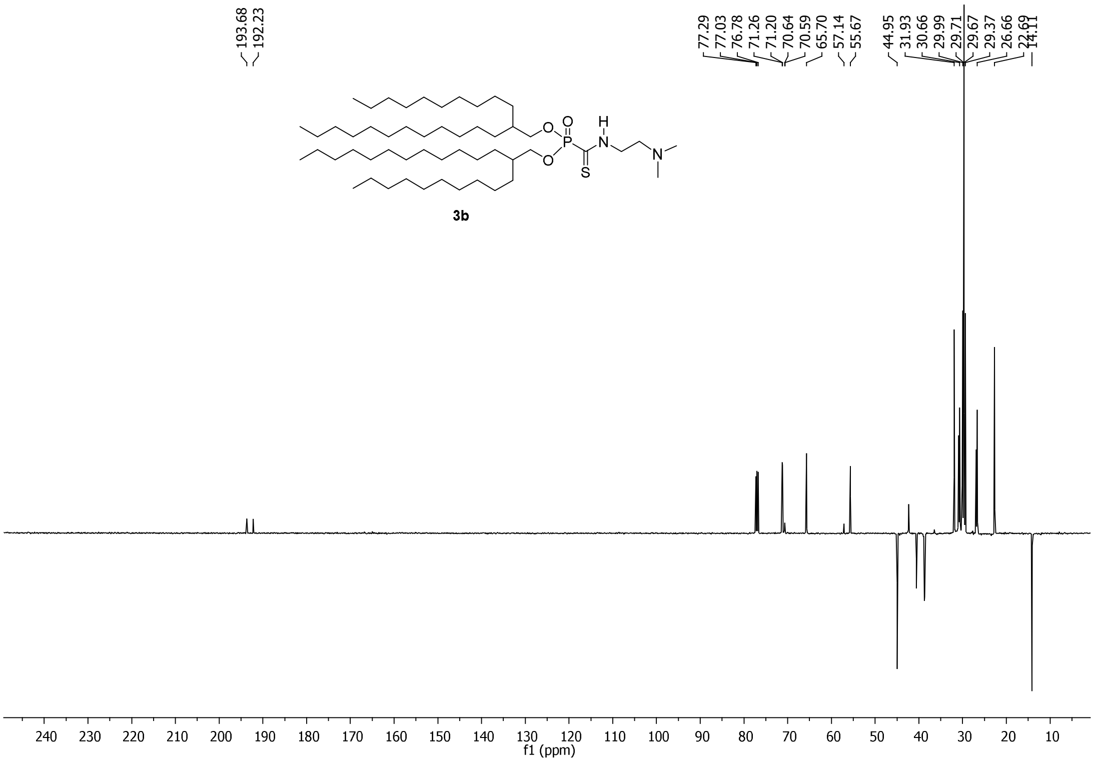

Figure S29. ^{13}C $\{^{1}H\}$ ($CDCl_3$) *of compound* ***3b***

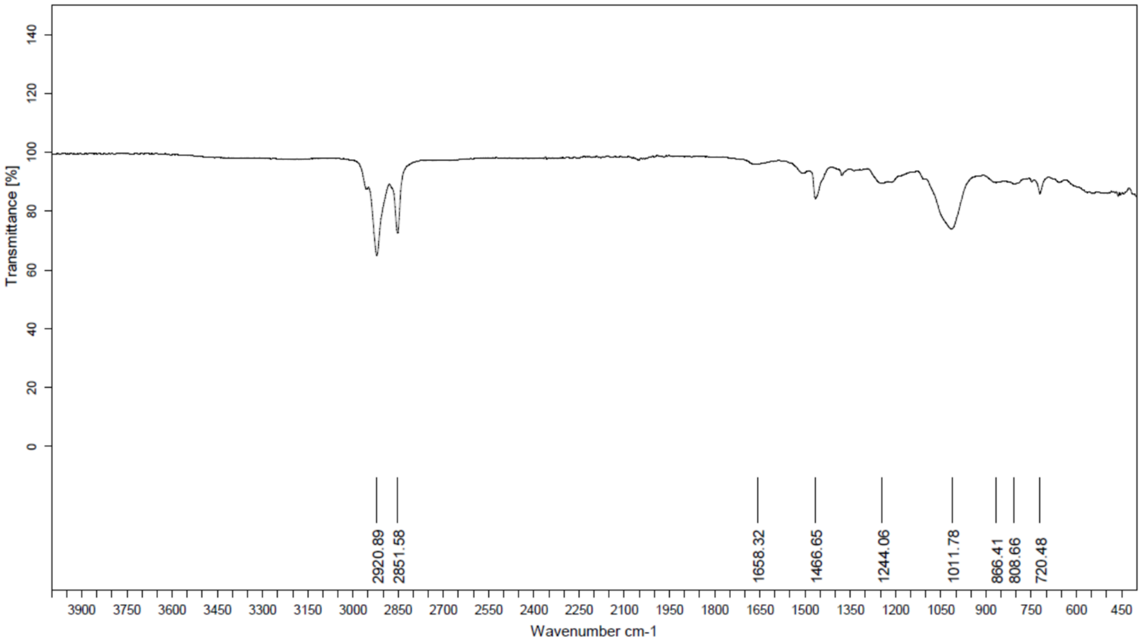

Figure S30. *IR (ATR) of compound **3b***

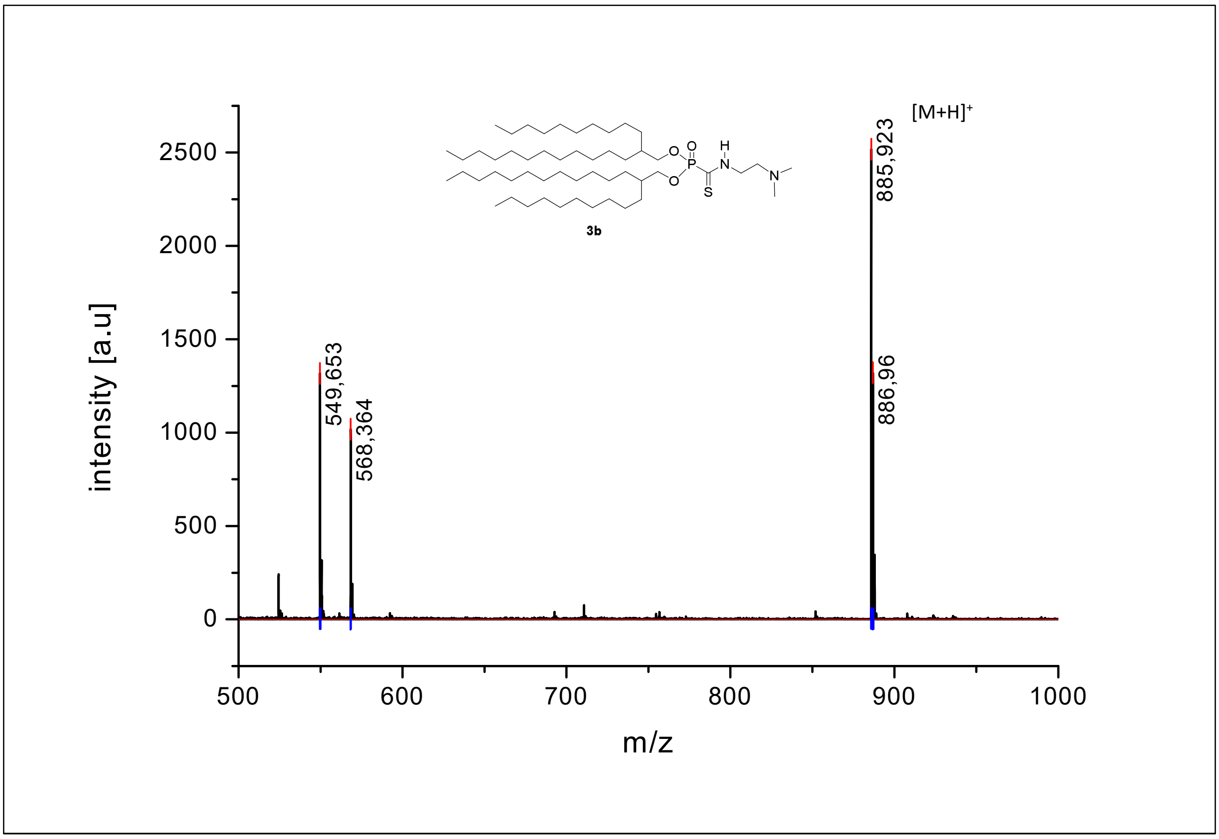

Figure S31**. MALDI-TOF (matrix: HCCA) of compound **3b

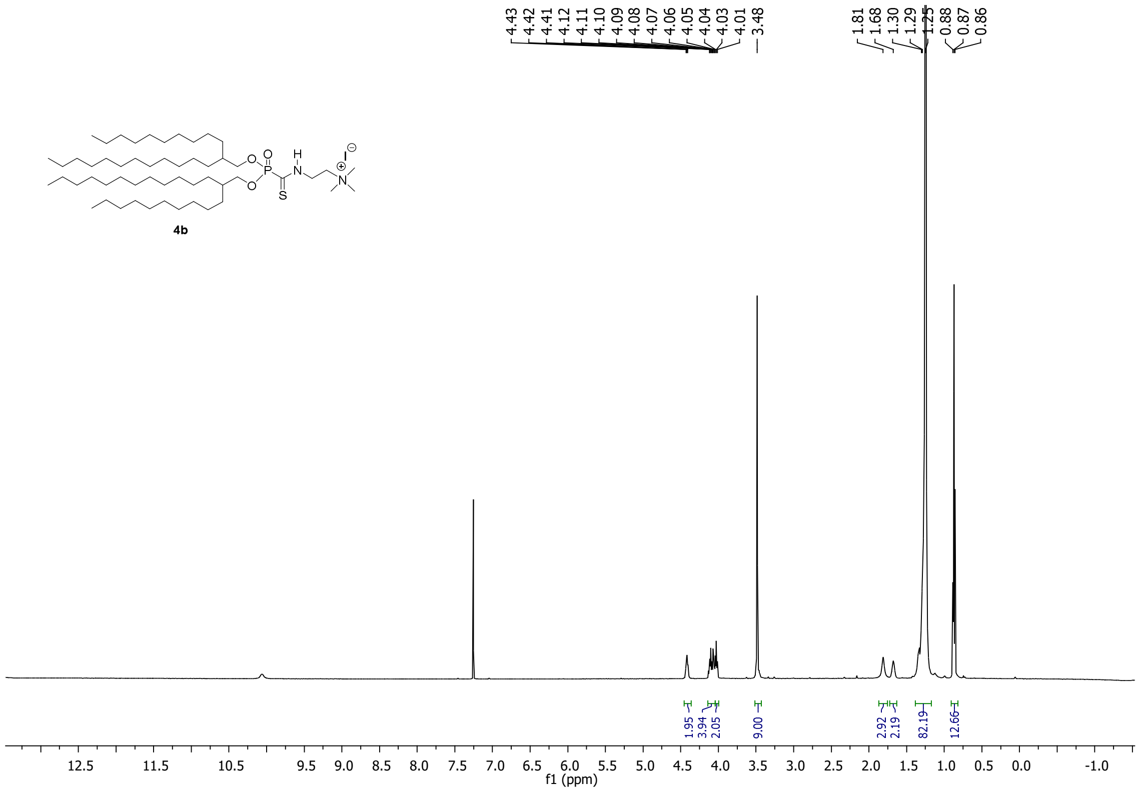

Figure S32**. ^{1}H NMR ($CDCl_3$) of compound **4b

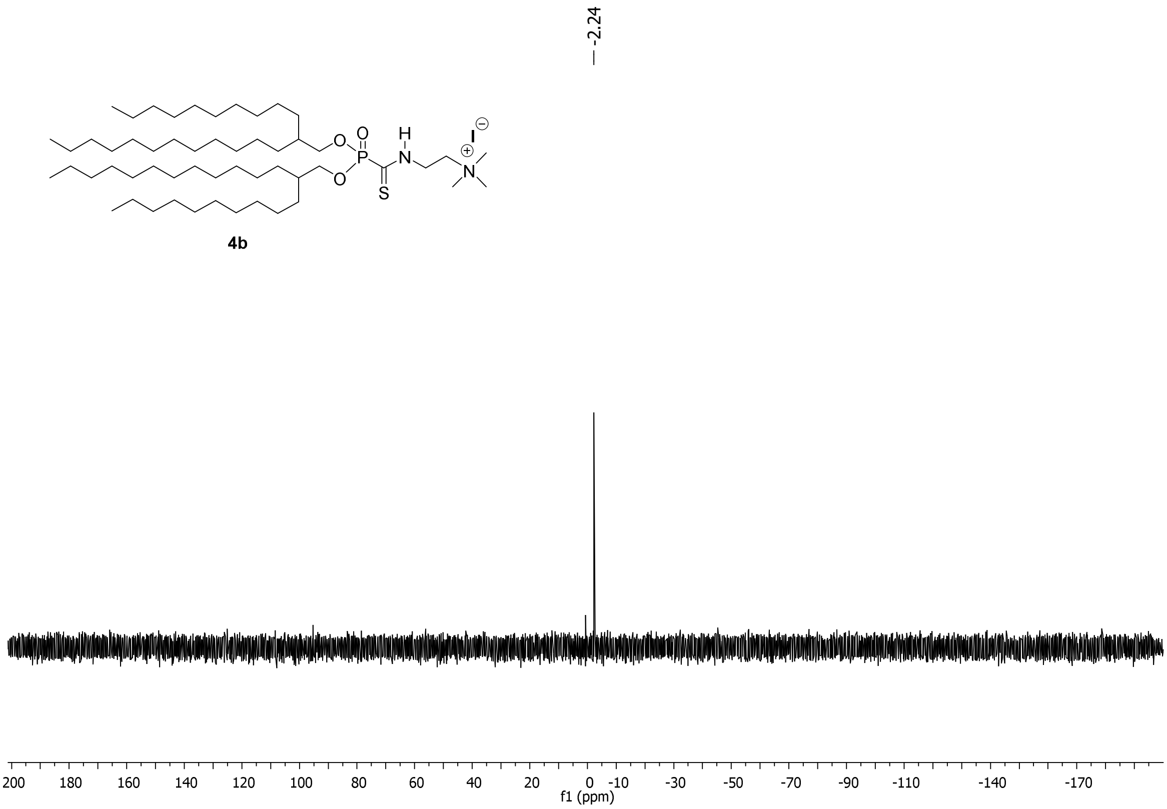

Figure S33. *^{31}P {^{1}H} (CDCl$_3$) of compound* ***4b***

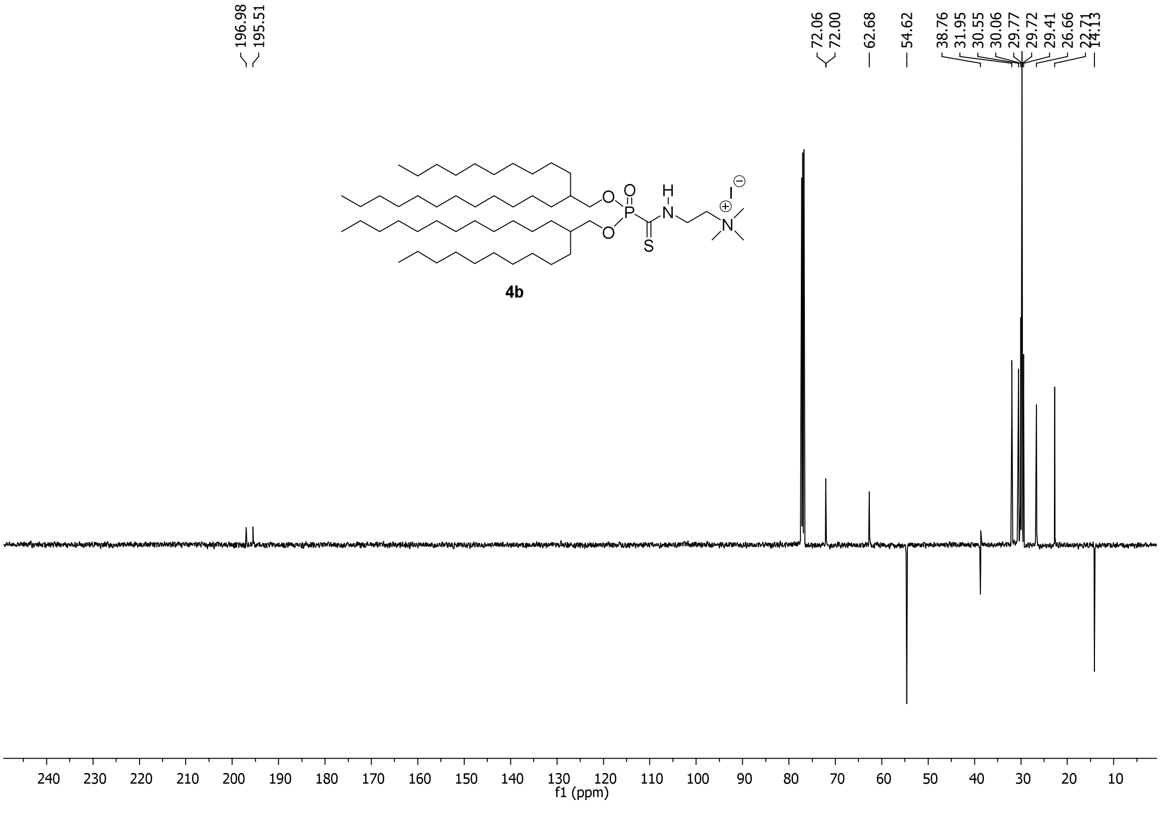

Figure S34. *^{13}C {^{1}H} (CDCl$_3$) of compound* ***4b***

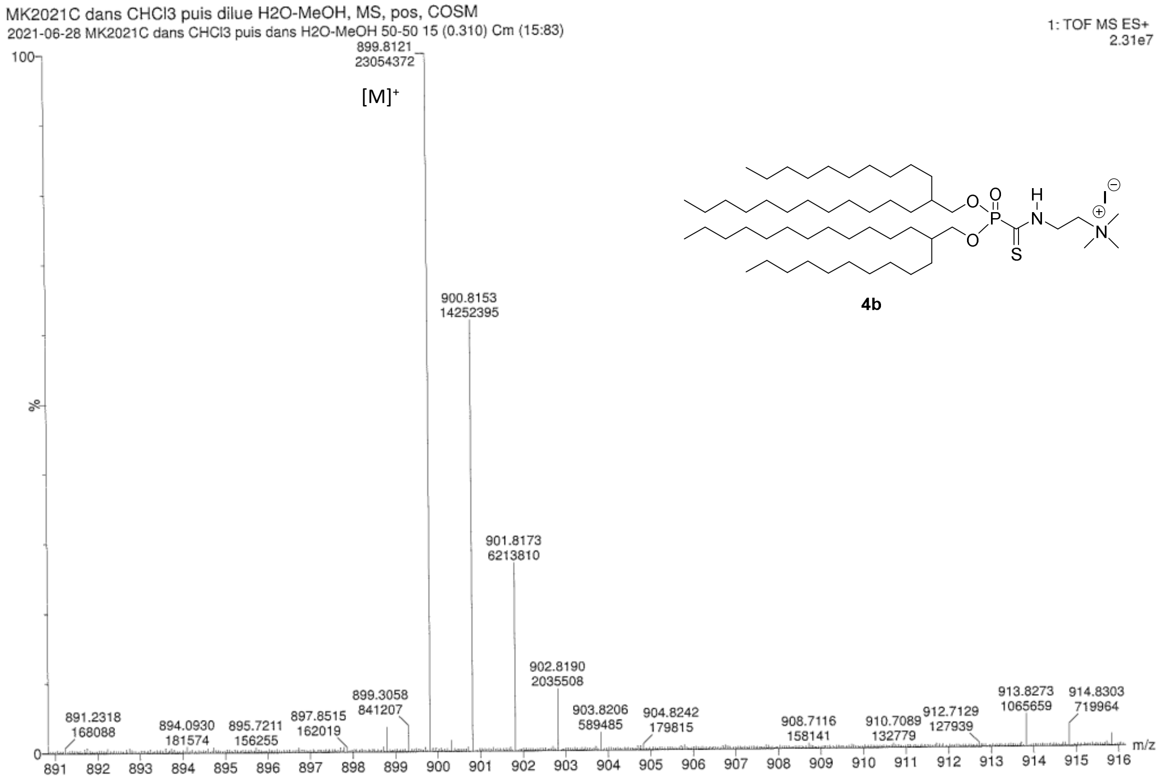

Figure S35. *ESI-qTOF MS of compound **4b***

2. Stability of the liposomes

	DLS measurements (t=0)			DLS measurements (t+ 5 months)[a]		
	Size (nm)	PDI	Zeta Potential (mV)	Size (nm)	PDI	Zeta Potentiel (mV)
**LF1**	227 ±41	0.46	61 ±1.7	129 ±4	0.49	56 ±1
**LF2**	194 ±16	0.44	79 ±0.6	163 ±7	0.52	52 ±2
**LF3**	229 ±15	0.33	57 ±0.8	154 ±3	0.27	49 ±1
**BSV36**	169 ±9	0.24	58 ±0.06	112 ±2	0.40	62 ±3
**KLN47**	126 ±7	0.33	28 ±1.16	143 ±36	0.40	52 ±1

a) The liposomes were store à 4-6°C for 5 months. Before the measurements, the liposomal solutions were sonicated for 10 minutes at 38°C.

Table S1*: Sizes and zeta potentials of the liposomes at t=0 and 5 months later.*

3. DLS and zeta potential of lipoplexes

Reference of the formulations	CR	Size (nm)	PDI	Zeta Potentiel (mV)
**BSV36**	0.7	155.7 +/- 1.704	0.230 +/- 0.015	-22.1 +/- 0.808
**BSV36**	1	185.5 +/- 0.6658	0.265 +/- 0.006	-13.3 +/- 1.01
**BSV36**	2	177.7 +/- 1.493	0.296 +/- 0.030	40.7 +/- 0.252
**BSV36**	4	136.7 +/- 1.422	0.295 +/- 0.032	47.7 +/- 0.208
**KLN47**	0.7	202.9 +/- 4.623	0.350 +/- 0.019	-20.7 +/- 1.07
**KLN47**	1	198.4 +/- 5.164	0.357 +/- 0.028	-22.2 +/- 0.208
**KLN47**	2	225.5 +/- 8.026	0.416 +/- 0.020	-8.35 +/- 0.514
**KLN47**	4	162.4 +/- 1.258	0.278 +/- 0.021	46.6 +/- 1.77
**LF1**	0.7	130.0 +/- 4.851	0.308 +/- 0.028	-17.8 +/- 0.643
**LF1**	1	138.3 +/- 0.3606	0.222 +/- 0.011	-19.7 +/- 0.874
**LF1**	2	133.3 +/- 0.7371	0.159 +/- 0.019	47.9 +/- 1.34
**LF1**	4	117.2 +/- 1.079	0.200 +/- 0.008	51.4 +/- 0.651
**LF2**	0.7	181.7 +/- 5.173	0.295 +/- 0.026	-20.0 +/- 6.92
**LF2**	1	188.3 +/- 0.8386	0.290 +/- 0.009	-18.6 +/- 2.40
**LF2**	2	200.2 +/- 1.665	0.265 +/- 0.004	-17.3 +/- 3.20
**LF2**	4	170.3 +/- 2.495	0.258 +/- 0.013	43.3 +/- 1.23
**LF3**	0.7	144.6 +/- 6.744	0.257 +/- 0.015	-13.0 +/- 0.611
**LF3**	1	152.1 +/- 3.753	0.275 +/- 0.038	-10.4 +/- 0.537
**LF3**	2	143.4 +/- 1.217	0.233 +/- 0.018	-11.0 +/- 0.100
**LF3**	4	142.4 +/- 1.973	0.209 +/- 0.004	-11.2 +/- 0.709

Table S2*: Sizes and zeta potentials of the lipoplexes at different charges ratios.*

4. Gel electrophoresis

Condition	***% complexed DNA***	***% Free DNA***
Naked DNA	0.00%	100.00%
LF1 CR 0.7	57.20%	42.80%
LF1 CR 1	74.01%	25.99%
LF1 CR 2	100.00%	0.00%
LF1 CR 4	100.00%	0.00%
LF2 CR 0.7	31.87%	68.13%
LF2 CR 1	35.00%	65.00%
LF2 CR 2	57.46%	42.54%
LF2 CR 4	100.00%	0.00%
LF3 CR 0.7	1.96%	98.04%
LF3 CR 1	4.70%	95.30%
LF3 CR 2	44.67%	55.33%
LF3 CR 4	94.43%	5.57%
BSV36 CR 0.7	45.36%	54.64%
BSV36 CR 1	57.09%	42.91%
BSV36 CR 2	96.28%	3.72%
BSV36 CR 4	97.63%	2.37%
KLN47 CR 0.7	16.25%	83.75%
KLN47 CR 1	19.75%	80.25%
KLN47 CR 2	69.34%	30.66%
KLN47 CR 4	96.27%	3.73%

Table S3 *: Fraction of complexed DNA and free DNA after complexation process with the cationic lipids determined by densitometry analysis on the electrophoresis gel.*

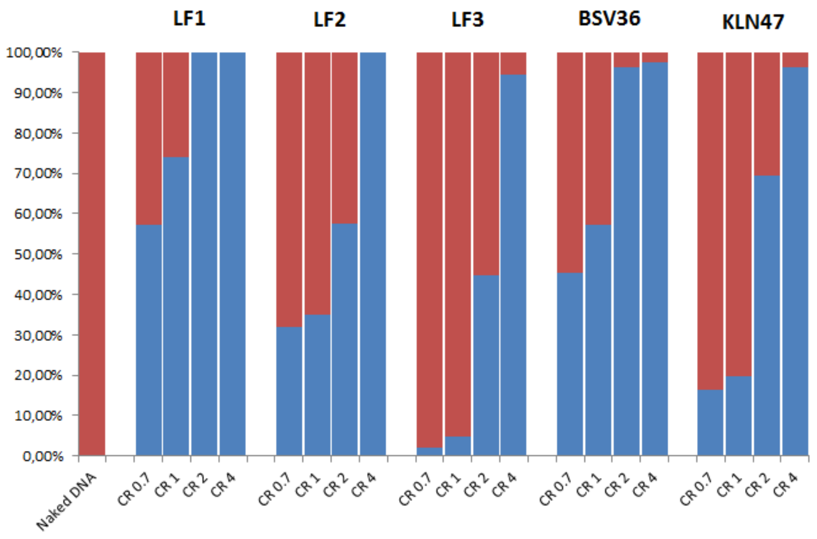

Figure S36 *Proportion of complexed and free DNA after complexation process with the cationic lipids determined by densitometry analysis on the electrophoresis gel. For each mixture tested, the fraction of free DNA is in red, while complexed DNA is in blue. Naked DNA was used to normalize each test as the quantity of DNA in all conditions was the same.*
